# The Hydrogenobyric Acid Structure Reveals the Corrin Ligand as an Entatic State Module Empowering B_12_ Cofactors for Catalysis

**DOI:** 10.1002/anie.201904713

**Published:** 2019-06-26

**Authors:** Christoph Kieninger, Evelyne Deery, Andrew D. Lawrence, Maren Podewitz, Klaus Wurst, Emi Nemoto‐Smith, Florian J. Widner, Joseph A. Baker, Steffen Jockusch, Christoph R. Kreutz, Klaus R. Liedl, Karl Gruber, Martin J. Warren, Bernhard Kräutler

**Affiliations:** ^1^ Institute of Organic Chemistry and Center for Molecular Biosciences University of Innsbruck 6020 Innsbruck Austria; ^2^ School of Biosciences University of Kent Canterbury CT2 7NJ UK; ^3^ Institute of General, Inorganic and Theoretical Chemistry, and Center for Molecular Biosciences (CMBI) University of Innsbruck 6020 Innsbruck Austria; ^4^ Department of Chemistry Columbia University New York USA; ^5^ Institute for Molecular Biosciences University of Graz Austria

**Keywords:** cobalamins, cobalt, synthetic biology, vitamins, X-ray structures

## Abstract

The B_12_ cofactors instill a natural curiosity regarding the primordial selection and evolution of their corrin ligand. Surprisingly, this important natural macrocycle has evaded molecular scrutiny, and its specific role in predisposing the incarcerated cobalt ion for organometallic catalysis has remained obscure. Herein, we report the biosynthesis of the cobalt‐free B_12_ corrin moiety, hydrogenobyric acid (**Hby**), a compound crafted through pathway redesign. Detailed insights from single‐crystal X‐ray and solution structures of **Hby** have revealed a distorted helical cavity, redefining the pattern for binding cobalt ions. Consequently, the corrin ligand coordinates cobalt ions in desymmetrized “entatic” states, thereby promoting the activation of B_12_‐cofactors for their challenging chemical transitions. The availability of **Hby** also provides a route to the synthesis of transition metal analogues of B_12_.

The unique structural^1^ and biosynthetic features^2^ of coenzyme B_12_ and its biological homologues raise fundamental questions concerning the evolution and selection of the corrin ligand,^3^ as well as the adoption of B_12_ cofactors into key metabolic roles across the three domains of life. The combined selection of the corrin macrocycle and of cobalt as the specific transition metal center for bio‐organometallic catalysis is an intriguing aspect of the B_12_ cofactors.^4^ The resistance of cobalt corrins against the removal of cobalt without concomitant destruction of the corrin ligand^5^ has made a study of cobalt‐free natural corrins a major scientific challenge.^6^ Consequently, despite the 40 years since vitamin B_12_ was prepared by total synthesis,^7^ the special partnership of the ligand and the cobalt ion of the natural B_12_ cofactors remains largely unexplored.4a

Two pathways for B_12_ biosynthesis have highlighted intriguing “ring contraction” steps^2^ that tailor the “coordination hole” of the tetrapyrrolic macrocycle to the effective size of cobalt ions.4a, 8 Surprisingly, B_12_’s own ligand, hydrogenobyric acid (**Hby**) (Figure 1), is not a biosynthetic intermediate in either of them.^2^ However, metabolic engineering of the B_12_ biosynthetic pathway has allowed the development of strategies to access metal‐free corrins by design.2b, 9 We recently reported recombinant *E. coli* strains that generated metal‐free corrins, such as hydrogenobyrinic acid a,c‐diamide (**HBAD**).^9^, ^10^ Normally, in the aerobic B_12_ biosynthetic pathway, **HBAD** is next chelated with cobalt.^2^ However, when grown in the absence of cobalt, some purple sulfur bacteria produce cobalt‐free corrinoids,^11^ including a compound tentatively identified as **Hby**,11b, 11c providing hope for the biological synthesis of **Hby**.2b Herein, we describe an engineered B_12_ biosynthesis pathway variant containing the enzyme CobQ for the effective preparation of **Hby**, and present a thorough analysis of the structure of this metal‐free corrin, which is critical for binding cobalt ions and for bestowing B_12_ biocatalysts with their exceptional reactivity.4a, 12

**Figure 1:**
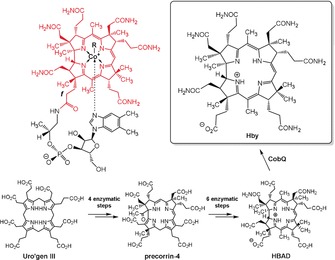
Structural formulae of hydrogenobyric acid (**Hby**) and of the cobalamins (**Cbls**) coenzyme B_12_ (*R*=5′‐adenosyl, **AdoCbl**), methyl‐cobalamin (R=CH_3_, **MeCbl**), vitamin B_12_ (R=CN, **CNCbl**) and cob(II)‐alamin (R=e^−^, **Cbl^II^**), and key steps of the designed de novo biosynthesis of **Hby**. A complete outline of the engineered biosynthesis of **Hby** is included in Figure S1 in the Supporting Information.

A pathway variant was explored for the biosynthesis of **Hby** by integrating *cobQ* from a purple sulfur bacterium11a into the existing repertoire of **HBAD** biosynthetic genes to generate a Hby‐operon in an *E. coli* strain called ED661. With the Hby‐operon integrated in the genome under the control of a T7 promoter, **Hby** was found to be excreted into the culture medium. A 4 L fermentation of this strain furnished 11.8 mg (12.8 μmol) of crystalline **Hby** (Figure 1 and Supporting Information, SI), providing an unprecedented opportunity to study a metal‐free natural corrin. When buffered to pH 5–7, and kept in the dark, aqueous solutions of **Hby** were found to be relatively stable at room temperature (at higher pH **Hby** was converted into “yellow corrinoids”).11a, 11b

In aqueous solution, **Hby** exhibited UV/Vis absorption11b with maxima at 270 nm, 330 nm, 499 nm and 524 nm, and emitted fluorescence with maxima at 552 and 609 nm (Figure 2), comparable to a natural “metal‐free red corrin”.^13^ The absorption and emission maxima (at 524 and 552 nm, respectively) position the lowest singlet excited state of **Hby** at 223 kJ mol^−1^ (for additional data see SI, Figure S2). NMR‐ and mass spectra (Figure 2 and see SI) established the structure of **Hby**. The signals of all H, C and N atoms of **Hby** were assigned via (^1^H,^1^H)‐homonuclear and (^1^H,^13^C)‐ and (^1^H,^15^N)‐heteronuclear single and multiple bond correlations. Two lowfield signals gave evidence for two “inner” H‐atoms at N2 and N4, specifying the structure of the cationic corrin ligand core in metal‐free **Hby**. Other NH tautomers, such as ^**1,3**^ **Hby** with “inner” H atoms at N1 and N3, were not detected (Figure 2). However, the HN2 and HN4 protons undergo unsymmetrical transannular H‐bonding with N1 and N3, detected with ^15^N‐labelled **Hby**, clarifying the question^6^ of the location and H‐bonding pattern of the “inner” H atoms in a natural metal‐free corrin. The H atoms H(N2) and H(N4) of **Hby** were also observed to interact mutually by NOE correlations and by an additional nonbonding through‐space interaction, diagnosed through substitution of either one of these H(N)s by D (see SI, Figure S4).

**Figure 2:**
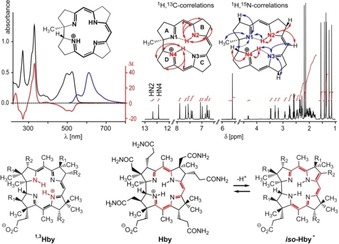
Spectra and structure of **Hby**. Top left: UV/Vis‐absorption (black trace), fluorescence emission (blue trace) and CD spectra (red trace) of **Hby**, recorded at 25 °C. Top right: 700 MHz ^1^H NMR spectrum of **Hby** in H_2_O/D_2_O (49:1) at pH 5 and correlations locating two “inner” HN protons at N2 and N4 and establishing their H‐bonds to N1 and N3. Bottom: The structure of **Hby** in water is represented best by the formula shown, while the tautomer ^**1**,**3**^ **Hby** (left) was not detected. Deprotonation of **Hby** generates an *iso*‐corrin anion, presumably ***iso*** **‐Hby^−^** (right), but not a “neutral” corrin (see SI for formulae); R_1_=CH_2_CONH_2_, R_2_=CH_2_CH_2_CONH_2_ in the formulae of ^**1,3**^ **Hby** and ***iso*** **‐Hby^−^**.

Both of the two “inner” H atoms are tightly bound by the corrin ligand, despite their fast exchange with water with rates of 21.9 s^−1^ (HN2) and 6.3 s^−1^ (HN4) at 308 K (SI, Figure S5). Indeed, the corrin moiety of **Hby**, a weak acid with p*K*_a_(**Hby**)=11.2,11b is deprotonated at the corrin periphery, presumably at C8 (Figure 2), as was first deduced by Eschenmoser and Fischli for the model corrin **HCor^+^** (formula and crystal structure in SI, Figure S6).^6^, ^14^, ^15^ Poignantly, a monoprotonated “neutral” corrin ligand^6^ remains elusive. These features of **Hby** are supported by DFT analyses, which are consistent with the experimentally found stable zwitterionic form of **Hby** with two unsymmetrical H‐bonds N1–HN2 and N3–HN4, support peripheral C8 as the most acidic position of **Hby** and indicate protomers of **Hby** with a single “inner” H atom, either at N4 or at N2, to be significantly less stable (see SI, Figures S7 and S8; Table S5).

**Hby** generated single crystals from H_2_O/MeCN at 5 °C, with space group *P*2_1_. X‐ray analysis revealed a pseudo‐C_2_‐symmetric helical arrangement of the core part of **Hby**, with similar structural features observed in the crystal as in solution (Figure 3 and SI, Figures S6 and S9). Electron density for two “inner” H atoms was located at N2 and N4, which were at a distance of only 2.27 Å from each other. The two H atoms are also close to N1 and N3 with distances of 1.91 Å and 2.06 Å, respectively, consistent with the NMR‐derived unsymmetrical H‐bonding. The distance between N2 and N4 of **Hby** is 3.97 Å, that is, about 0.3 Å longer than that between N1 and N3 (3.67 Å). By contrast, in **HCor^+^**, the “inner” H atoms are located at N1 and N3.^6^, ^14^, ^15^ However, H(N1) of **HCor^+^** undergoes H‐bonding interactions to an EtOH molecule, giving the C4–C5 bond of **HCor^+^** a 24.8° twist.^6^, ^15^ In both, **Hby** and **HCor^+^**, the “inner” H atoms break the inherent C_2_ symmetry of the corrin core, contrasting with the situation in the more regularly structured cobalt corrins and in the “expanded”, symmetrical porphyrins.^16^

**Figure 3:**
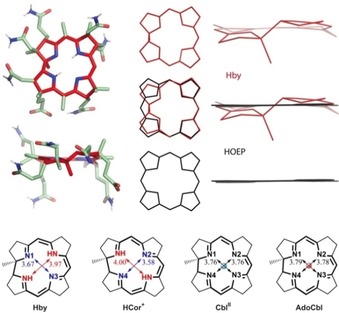
The ring‐contracted corrin ligand is a uniquely skewed helix. Top left: Two projections of the crystal structure of **Hby** (color coding: carbons of corrin core: red; of substituents: green; nitrogens: blue; oxygens: red; hydrogens: white). Top center and right: Projections of core structures of **Hby** (red), of octaethyl‐porphyrin (**HOEP**, black) and their superposition (middle). Bottom: Core structures of the metal‐free corrins **Hby** and **HCor^+^**,^14^, ^15^ and of the Cbls **Cbl^II^** and **AdoCbl**, in which effects of “inner” H atoms or of Co ions on the lengths of diagonals are highlighted.

The corrin **Hby** features a coordination hole with an average diameter of 3.83 Å, indicating an effective ring contraction of roughly 0.3 Å, compared to octaethylporphyrin (**HOEP**).^16^ Hence, the effective coordination radius in **Hby** (1.916 Å) is close to the average equatorial (Co–N) bond in **AdoCbl** (1.897 Å),^17^ **MeCbl** (1.898 Å)^18^ and in **Cbl^II^** (1.88 Å).^19^ At first sight, the corrin ligand appears to be well adapted for coordination of Co^III^ and Co^II^ ions.^17^, ^18^, ^19^ However, the corrin‐specific *trans* junction between rings A and D imposes a distinctly helical structure.^1^ Consequently, the four chelating N atoms of the corrin macrocycle of **Hby** represent a screw‐like coordination hole, leading to a coordinative misfit for cobalt ions that is particularly strong for Co^III^.

The mutual conformational adaptation of the corrin ligand and the coordinated cobalt ions was evaluated by two structure parameters: i) The corrin helicity *h* of the innermost coordination space of the corrin ligand provided by the four corrin nitrogen atoms, defined by the dihedral angle N1‐N2‐N3‐N4 (see Figure 4). In the metal‐free corrin **Hby** it amounts to *h*(**Hby**)=12.9°. Co^III^ corrins feature strongly reduced *h* values, e.g., *h*(**AdoCbl**)=3.5° and *h*(**MeCbl**)=4.6°. Hence, the ligand is strongly flattened by Co^III^ binding in **AdoCbl** and **MeCbl**. On the other hand, the four‐coordinate Co^II^ center (Cbl^II^ACA) of the human adenosyl‐transferase ACA fits the corrin ligand better, displaying *h*(Cbl^II^ACA)=8°.^20^ Five‐coordinate Co^II^ corrins display lower intermediate levels (see Figure 4). ii) The interplanar angle *φ*, which concerns the equatorial coordination sphere at the cobalt center, indicating coordinative strain in cobalt corrins when deviating from 0° (see Figure 4 and SI for details). The reference value of **Hby** is *φ*=13.5°. In Cbl^II^ACA *φ*=17°, in the two Co^II^ corrins, **Cbl^II^** and **Cbin^II^** 21 *φ* is 12.5°, respectively 7.6°. In Co^III^ corrins, like **AdoCbl** and **MeCbl**, *φ* is only 4–5°. Hence, *h* and *φ* decrease in a roughly correlated fashion from **Hby** to Co^II^ and to Co^III^ corrins, indicating significant directional coordinative misfit in Co^III^ corrins.

**Figure 4:**
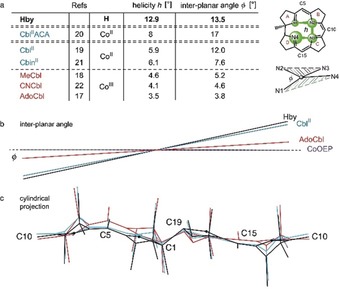
Structural characteristics of the coordinative interaction between cobalt ion and corrin ligand in B_12_ derivatives. a) Table with data describing the mutual adaptation of cobalt ions and the natural corrin ligand, expressed by the corrin helicity *h* (the calculated dihedral angle N1‐N2‐N3‐N4) and by the angle *φ* between the planes [N1‐cobalt‐N4] and [N2‐cobalt‐N3] (see drawings at right). In the order **Hby**, Co^II^ corrins and Co^III^ corrins, *h* and *φ* decrease both in a roughly correlated fashion. b) The interplanar angle *φ* is large in helical **Hby** and in **Cbl^II^**, but strongly reduced in **AdoCbl**. In four‐coordinate Co^II^ and six‐coordinate Co^III^ porphyrins (CoOEP) *φ*=ca. 0°.^16^ c) Cylinder projections of the structures of **Hby**, **AdoCbl** and **Cbin^II^**, highlighting conformational differences in the corrin ligand. The conformation of **Hby** (black trace) is largely retained in the Co^II^ corrin **Cbin^II^** (blue trace), contrasting with its stronger adaptation to Co^III^ binding in **AdoCbl** (red trace).

The structural analysis of the helical corrin ligand **Hby** of B_12_ derivatives has revealed key elements helping to “demystify vitamin B_12_”.^3^, ^4^ It has confirmed the postulated “fit”^3^, ^4^, ^8^ of the “ring‐contracted” corrin ligand **Hby** to the size of Co^III^ and Co^II^ ions (in **AdoCbl** and **Cbl^II^**). However, the corrin ligand **Hby** is distinctly helical, dissatisfying the octahedral coordination preference of Co^III^ centers, while better meeting the requirements of Co^II^ and Co^I^ ions (Figures 4 and 5). The inferior accommodation of Co^III^ over Co^II^ centers implies a previously overlooked coordinative strain for Co^III^ corrins that promotes homolytic (Co–C) bond cleavage. This effect is crucial for the homolysis of **AdoCbl** to **Cbl^II^** in the B_12_‐dependent radical isomerization reactions.4c, 23 The same type of strain also activates the cobalt‐bound methyl group of **MeCbl** for abstraction by radicals^24^ in B_12_‐dependent radical SAM enzymes.^25^ A similar strain decrease may also accompany the heterolytic abstraction of the cobalt‐bound methyl of **MeCbl** by nucleophiles in B_12_‐dependent enzymatic methyl group transfer, producing Co^I^ cobalamin.^26^ In the critical adenosyl‐transferase ACA, an unstable four‐coordinate form of **Cbl^II^** (Cbl^II^ACA)^20^ undergoes the reduction to the four‐coordinate Co^I^ species. Such essential four‐coordinate Co^II^ and Co^I^ forms, which are hard to generate metabolically,25b, 27 appear to be well accommodated by the helical coordination hole of the corrin ligand. Since Co^I^ corrins are not structurally characterized, model DFT calculations were used. They indicate a reduction of coordinative strain, by about 7 kJ mol^−1^, for the transition from six‐coordinate Co^III^ to four‐coordinate Co^I^ ions, when bound by four N atoms in a nonplanar arrangement, as in **Hby**. The analogous Co^III^‐to‐Co^II^ transition experiences a strain decrease of about 10 kJ mol^−1^ (SI, Figure S10). Hence, the inherently helical corrin ligand acts as a “Procrustean bed” that destabilizes Co^III^ centers towards loss of axial ligands and formation of Co^II^ or Co^I^ forms, enhancing catalysis by the B_12_ cofactors.

**Figure 5:**
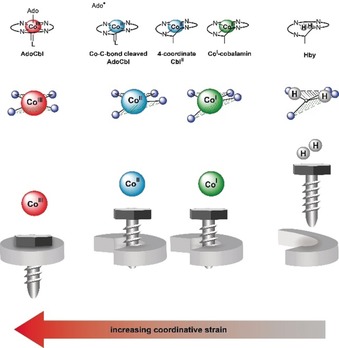
The helical corrin ligand binds cobalt centers in a strained state, promoting the cleavage of axial bonds and formation of reduced corrinoids. This is symbolized at the top for the Co^III^ corrin **AdoCbl** (before and after Co–C bond cleavage), for four‐coordinate Co^II^ and Co^I^ cobalamin, and for **Hby**. Middle and bottom: The corrin ligand is flattened and interplanar angle *φ* decreased most strongly at Co^III^ centers, less at Co^II^ and Co^I^ ions. Both parameters indicate an increasing misfit and strain in the series Co^I^/Co^II^ and Co^III^ corrins; numerical data for *h* and *φ* are collected in Figure 4.

The previously unrecognized role of the flexible helical corrin ligand in activating organometallic Co^III^ corrins for catalysis classifies the B_12_ cofactors **AdoCbl** and **MeCbl** as “entatic state” molecules. The term “entatic” state was initially applied to proteins with metal centers bound in a strained coordination sphere to lower activation barriers for enzyme catalysis.^28^ Herein, we infer that cobalt corrins have been selected4a since they represent “entatic state” complexes in which ligand‐imposed strain activates Co^III^ centers for catalysis. A related situation exists in coenzyme F_430_, a Ni corphinoid, in which radial strain results from a misfit between the size of the coordinated Ni ions and the porphyrinoid macrocycle.4a, 29

The availability of the metal‐free **Hby** has also opened the door to the direct preparation of transition metal analogues of the cobalamins, the “metbalamins” (**Metbl**s), a “Holy Grail” of bioinorganic chemistry.^6^, ^9^, 11b, 11c, 30 Hence, **Hby** has served as an effective starting material for the synthesis of transition metal B_12_ analogues, to be reported shortly. As described with **AdoRhbl**, the Rh^III^ analogue of **AdoCbl**,^9^ suitably structured **Metbl**s hold a significant potential as “antivitamins B_12_”, in biological imaging or as novel antibiotics.^31^ The exciting prospect of investigations with transition metal complexes of the skewed corrins will interest experimental scientists and theoretical chemists alike.

## Experimental Section

CCDC 1881269 (**Hby**, see SI) contains the supplementary crystallographic data for this paper. These data can be obtained free of charge from The Cambridge Crystallographic Data Centre.

## Conflict of interest

The authors declare no conflict of interest.

## Supporting information

As a service to our authors and readers, this journal provides supporting information supplied by the authors. Such materials are peer reviewed and may be re‐organized for online delivery, but are not copy‐edited or typeset. Technical support issues arising from supporting information (other than missing files) should be addressed to the authors.

Supplementary
