# Supplementary material for: The Hydrogenobyric Acid Structure Reveals the Corrin Ligand as an Entatic State Module Empowering B12 Cofactors for Catalysis
Source: Angew Chem Int Ed Engl. 2019 Jun 26;58(31):10756–60. doi: 10.1002/anie.201904713 (PMC6771967; doi:10.1002/anie.201904713)
Supplement: Supplementary file 1 — Supplementary [file ANIE-58-10756-s001.pdf]

Angewandte Chemie
Eine Zeitschrift der Gesellschaft Deutscher Chemiker

Supporting Information

The Hydrogenobyric Acid Structure Reveals the Corrin Ligand as an Entatic State Module Empowering B_{12} Cofactors for Catalysis

Christoph Kieninger+, Evelyne Deery+, Andrew D. Lawrence+, Maren Podewitz+, Klaus Wurst, Emi Nemoto-Smith, Florian J. Widner, Joseph A. Baker, Steffen Jockusch, Christoph R. Kreutz, Klaus R. Liedl, Karl Gruber, Martin J. Warren, and Bernhard Kräutler**

anie_201904713_sm_miscellaneous_information.pdf

Author Contributions

C.K. Data curation: Lead; Investigation: Lead; Writing—Original Draft: Equal; Writing—Review & Editing: Supporting
E.D. Methodology: Lead; Resources: Equal
A.L. Investigation: Lead; Methodology: Equal; Resources: Equal
M.P. Funding acquisition: Equal; Methodology: Lead.

Supporting Information

Table of Contents

1. Materials 2

2. Instruments 2

3. Construction of bacterial strains and biosynthetic preparation of hydrogenobyric acid (Hby).. 3

4. Purification and crystallization of Hby........ 3

5. Biosynthetic preparation of ^{15}N-enriched Hby (^{15}N-Hby) 5

6. Determination of the exchange rates of inner corrin and amide protons by NMR 6

7. Crystal structure of Hby by single-crystal X-ray diffraction........ 7

7.1. Structural parameters derived from the X-ray crystal structure 7

8. Computational methodology 8

9. Supporting Figures 9

10. Supporting Tables........ 24

11. References 29

1. Materials

Methanol (MeOH), acetonitrile (MeCN), HiPerSolv Chromanorm, sodium hydroxide and acetic acid (HOAc) p.A. from VWR chemicals; sodium acetate (NaOAc), ammonium acetate (NH_4OAc), sodium tetrafluoroborate, p.A. from Fluka; methyl tert-butyl ether (MTBE) from Riedel de-Haen; tetrafluoroboric acid, sodium dihydrogenphosphate (NaH_2PO_4), disodium hydrogenphosphate (Na_2HPO_4), magnesium sulfate ($MgSO_4$), calcium chloride ($CaCl_2$), Thiamine, ammonium chloride (NH_4Cl) from Sigma Aldrich (UK) or from Fluka; acetone and toluene from Donauchemie; water purified by reversed osmosis and MilliQ academic system; D_2O 99.96%D from Eurisotop; LiChroprep RP18 (25-40 µm) from Merck; Sep-Pak® C18 cartridges (various sizes, conditioned with 20 mL MeOH and 60 mL H_2O prior to use) from Waters; Tryptone, Yeast extract from Oxoid; isopropyl-β-thiogalactopyranoside (IPTG) and 4-(2-hydroxyethyl)-1-piperazineethanesulfonic acid (HEPES) buffer from Melford; sodium chloride, glycerol and D-glucose from Fisher. ^{15}N-ammonium sulfate ($(^{15}NH_4)_2SO_4$ from Cambridge Isotope Laboratory. Amberlite XAD 2 from Supelco; nickel chelating sepharose from GE Healthcare.

2. Instruments

UV-Vis: Agilent Cary 60. CD: Jasco J-715 spectro-polarimeter. Fluorescence: HORIBA Jobin Yvon Fluorolog-3 (steady-state fluorescence spectra); Edinburgh Analytical Instruments OB920 (fluorescence lifetimes). Singlet oxygen phosphorescence: HORIBA Jobin Yvon, modified Fluorolog-2 spectrometer in conjunction with a NIR sensitive photomultiplier tube (Hamamatsu, H102330A-45). A 450 W Xe was used for steady-state excitation to record singlet oxygen phosphorescence spectra and a Spectra Physics GCR-150-30 Nd:YAG laser (355 nm, ca. 5 mJ per pulse, 5 ns) was used for pulsed excitation to collect phosphorescence decay traces at 1270 nm. NMR: 600 MHz Bruker Avance II+ with Prodigy TCI^{TM} probe, 700 MHz Bruker Avance 4 Neo with TXI probe; all NMR-spectra were measured for solutions of **Hby** in 10 mM aqueous Na-phosphate buffer pH 5; ^1H reference to $\delta(H_2O) = 4.75$ ppm, ^{13}C external reference on 2,2-dimethyl-2-silapentane-5-sulfonic acid (DSS), ^{15}N indirect reference on NH_3 (l); ESI-HR-MS: Thermo Scientific LTQ-Orbitrap XL, (+)-ion mode, 4.5 kV. HPLC: Hitachi Elite LaChrom, L2130 pump, L245 diode array detector; Dionex Ultimate 3000, variable wavelength detector; column: YMC-Triart –C18, 250x4.7 mm, S-5 µm, 12 nm, TATA12S05-2546WT; solvent composition: A: 10 mM aqueous NH_4OAc pH 7, B= MeOH; 8% to 95%B 0-40 min, 95% B 40-44 min, 95% to 8%B 44-45 min, flow= 1 mL/min. RP18-MPLC: Büchi C-605 pump module (binary) flow≈10 mL/min, self

packed RP18 column (l=230 mm, Ø=26 mm, column volume (CV)=122 mL, ~100 g LiChroprep RP18).

3. Construction of bacterial strains and biosynthetic preparation of hydrogenobyric acid (Hby)

The *cobQ* gene from a purple sulphur bacterium was cloned to allow its co-expression with the genes for **HBAD** [1] synthesis (*cobAIGJFMKLHB*) thereby generating a **Hby** operon. This was achieved through the construction of strain ED661, (*E. coli* with the **Hby** operon integrated in the genome under the control of the T7 promoter), which was grown in a 4 L fermenter (Sartorius) in 2YTNN medium (64 g tryptone, 40 g yeast extract, 20 g NaCl, 8 g glucose, 160 g glycerol, 11 g Na_2HPO_4, 2.7 g NaH_2PO_4) at 28 °C for a total of 50 h. The culture was induced with IPTG (0.4 mM) 8 h after inoculation. Interestingly, cell extracts of this strain were found to contain a mixture of partially (tri, tetra, penta) and fully (hexa) amidated derivatives of hydrogenobyrinic acid (**HBA**).[2] However, the culture supernatant was found to contain exclusively the fully amidated form. Hence, purification of **Hby** was performed with the culture supernatant following centrifugation, which was adjusted to pH 4.5 and applied to a Amberlite XAD 2 column (1 kg). The column was washed with water and then increasing stepwise methanol gradient to 30%. Elution of **Hby** was achieved with 50% methanol. The methanol was removed under vacuum and the product applied to an RP18 column equilibrated in water. The column was washed with 20% methanol and eluted with 30% methanol. Removal of the solvent afforded a crude product.

4. Purification and crystallization of Hby

All operations were carried out under low light conditions. The crude wet **Hby** extract (about 8 g, containing ~121 µmol **Hby**), was dissolved under sonication in ~50 mL H_2O and applied on the RP18-MPLC column, which was washed with 5 column volumes (CV) 10 mM aqueous NaOAc pH 6, 2.5 CV 5% (*v*/*v*) followed by 5 CV 10% (*v*/*v*) MeOH in 10 mM aqueous NaOAc pH 6. The orange band of **Hby** was eluted with 60% (*v*/*v*) MeOH in 10 mM aqueous NaOAc pH 6. The eluate was concentrated under reduced pressure at 50 °C. The concentrate was diluted with H_2O to ~20 mL and separated into 4 portions. For precipitation of proteins, 45 mL MeOH were added to each portion and centrifuged at 5.5 krpm for 30 min at room temperature (RT). The supernatant was decanted, the pellet re-dissolved in 3-5 mL H_2O, 45 mL MeOH added and centrifuged at 5.5 krpm for 30 min at RT. The MeOH-precipitation procedure was repeated twice. The supernatants were

combined and evaporated on the rotary evaporator at 50 °C. The residue was dissolved in 20 mL MeOH and precipitated with 250 mL MTBE. The precipitate was filtered off by suction. The solid was dissolved in 500 mL 10 mM aqueous NaOAc pH 6 (~82 µmol **Hby**) and loaded on the RP18-MPLC column. The **Hby** sample was separated using a continuous solvent gradient in 10 h from 0 to 60 % (*v*/*v*) MeOH in 10 mM aqueous NaOAc pH 6. Fractions containing **Hby** with enhanced purity were combined concentrated to ~200 mL and diluted with 10 mM aqueous NaOAc pH 6 to 500 mL. The orange solution was loaded on the RP18-MPLC column and separated in 10 h with 0 to 60 % (*v*/*v*) MeOH in 10 mM aqueous NaOAc pH 6. The **Hby** containing fractions were combined and divided into 2 portions. Each was diluted with H_2O to ~120 mL and loaded on a Sep-Pak® C18plus long cartridge. The adsorbates were washed with 60 mL H_2O. The adsorbates were eluted with 4 mL MeOH and the solutions were frozen in N_2(l). The solvents were evaporated under high vacuum (HV, ≤5*10^{-5} bar, RT) over night. The residues were dissolved in 400 µl H_2O at 30 °C under sonication and each precipitated by the addition of 4 mL MeCN. The precipitates were washed with MeCN, dissolved in 600 µl MeOH and precipitated with 4 mL MTBE. The semi-crude sample of **Hby**-precipitates dissolved in 1 mL H_2O and lyophilized. The precipitated **Hby** (~70 µmol ≊61 mg) was dissolved in 50 ml 10 mM aqueous NaOAc pH 6 and loaded on the RP18-MPLC column. The adsorbed **Hby** was purified using 8% (*v*/*v*) MeCN in 10 mM aqueous NaOAc pH 6 for 5.5 h, 9 % (*v*/*v*) MeCN for 3.5 h and 11 % (*v*/*v*) MeCN. The fractions of pure **Hby** were combined and diluted with H_2O to 1400 mL (~58 µmol). The **Hby** sample was adsorbed on a Sep-Pak® C18 20cc vac cartridge, washed with 200 mL 0.1% aq. HOAc and eluted with 0.1% mehtanolic HOAc. The solution was concentrated at 35 °C to ~70 mL, diluted with H_2O to 300 mL and loaded on two Sep-Pak® C18 plus long cartridges. The adsorbed **Hby** was washed with 20 mL H_2O, 20 mL 100 mM aq. $NaBF_4$ pH 6 followed by 20 mL H_2O and eluted with 3 mL MeOH containing 100 µM $NaBF_4$. The methanolic **Hby** solutions were combined, frozen in N_2(l) and evaporated under HV. The purified **Hby** was crystallized from H_2O/MeCN at 5±3 ˚C. The crystals were washed with MeCN and dried, yielding 11.24 mg (12.8 µmol) crystalline **Hby**.

UV/Vis (c=31.5 µM in 10 mM aq. sodium phosphate pH 5, RT): λ_{max} [nm] (lg ε) = 524 (4.21), 499 (4.19), 472 (sh, 3.95), 392 (3.57), 376 (3.64), 330 (4.61), 319 (sh, 4.42), 270 (4.43), 233 (4.01), 228 (4.01)

CD (c=63.1 µM in 10 mM aq. sodium phosphate pH 5, 293K): $\lambda_{max/min}$ [nm] (±Δε [l*mol^{-1}*cm^{-1}]) = 522 (-10.8), 496 (-9.9), 394 (1.4), 363 (0.1), 330 (52.3), 320 (sh, 30.9), 283 (sh, -11.2), 271 (-21.0), 253 (sh, -9.9), 233 (13.0); λ_0 [nm] = 409, 296, 243

Fluorescence (EtOH, RT): emission (λ_{ex} = 498 nm): λ_{max} [nm] 525, 609; Φ_f = 0.18; τ_f = 3.3±0.2 ns; excitation (λ_{em} = 606 nm): λ_{max} [nm] 270, 328, 496, 525; (EtOH glass, 77K): emission

(λ_{ex} = 498 nm): λ_{max} [nm] 538, 595, 643; τ_f = 7.4±0.1 ns; excitation (λ_{em} = 606 nm): λ_{max} [nm] 328, 496, 525.

HR-MS (MeOH, 4.5kV): m/z (I [%]) = 875.513 (100, $[C_{45}H_{66}N_{10}O_8+H]^+ \mathrel{\widehat{=}} [M+H]^+$), 876.515 (53), 877.518 (15), 878.522 (3)

NMR: chemical shift of assignment of ^{1}H (66/66), ^{13}C (45/45) and ^{15}N (8/10) via 700 MHz ^{1}H, (^{1}H, ^{1}H)-COSY, (^{1}H, ^{13}C)-HSQC, (^{1}H, ^{13}C)-HMBC, (^{1}H, ^{1}H)-ROESY and (^{1}H, ^{15}N)-HSQC spectra in H_2O/D_2O 49:1, 10mM sodium phosphate pH 5 (see Supporting Table 1.).

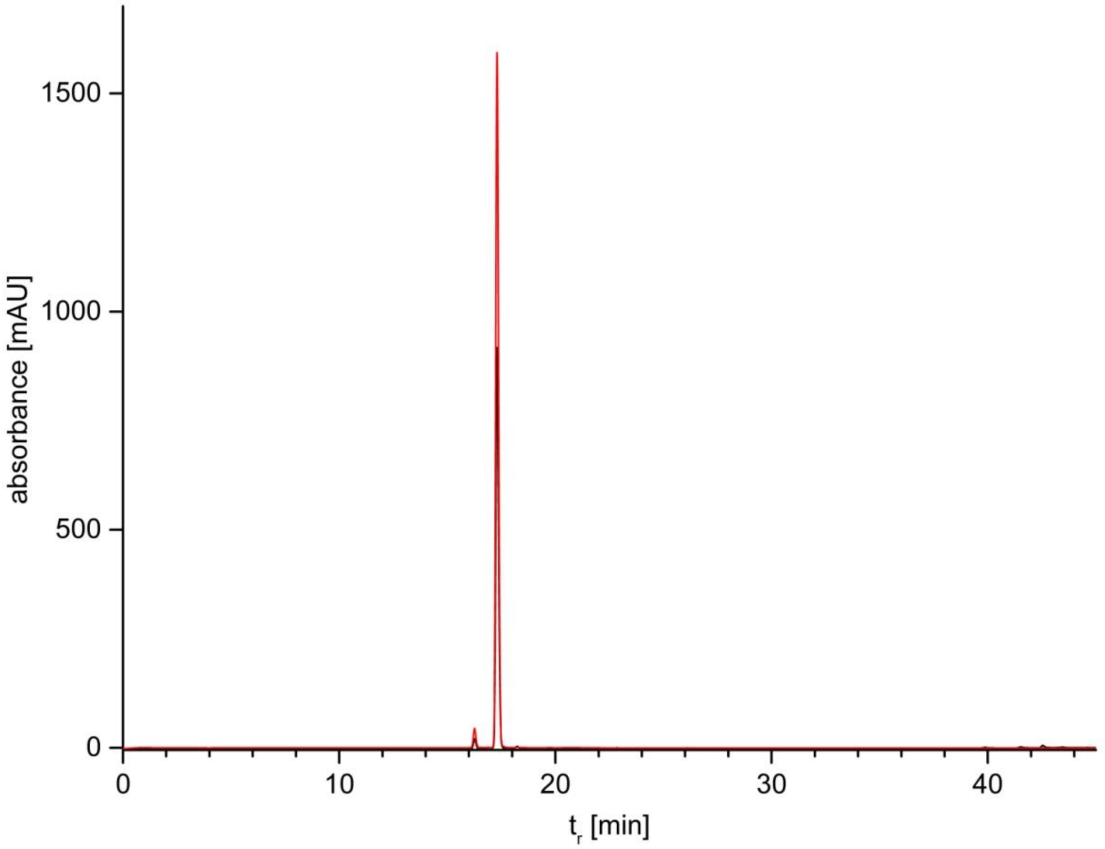

HPLC chromatogram of crystallized **Hby** (black trace: λ=280nm; red trace: λ=520nm); Dionex Ultimate 3000, variable wavelength detector; column: YMC-Triart –C18, 250x4.7 mm, S-5 µm, 12 nm; solvent composition: A: 10 mM aqueous NH_4OAc pH 7, B= MeOH; 8% to 95%B 0-40 min, 95% B 40-44 min, 95% to 8%B 44-45 min, flow= 1 mL/min.

5. Biosynthetic preparation of ^{15}N-enriched Hby (^{15}N-Hby)

^{15}N-**Hby** was synthesized using ^{15}N-enriched media, purified by affinity chromatography using BtuF [3, 4]. Strain ED661 was grown in a 4 L fermenter containing 24 g Na_2HPO_4, 12 g KH_2PO_4, 2 g NaCl, 80 g glycerol, 4 g D-glucose, 4 g yeast extract, 8 mL $MgSO_4$ (1 M), 4 mL $CaCl_2$

(0.1 M), 4 mL Thiamine (1 mg/mL), 1 g $(^{15}NH_4)_2SO_4$ and 3 g NH_4Cl. The culture was incubated at 28 °C for a total of 50 h, IPTG was added after 6 h. Strain ED-BtuF (overexpressing the B_{12}/corrinoid binding protein, BtuF with an N-terminus His-tag) was grown in 2YTNN. 32 L were grown in order to provide enough BtuF to purify the sample of ^{15}N-**Hby** from 4 L of the Hby-producing strain. ED-BtuF cell pellets were sonicated and purified with nickel chelating sepharose. The supernatant of the Hby culture was applied onto the nickel-His-BtuF column. The column was washed with 20 mM HEPES buffer, pH 7.5, containing 100 mM NaCl. ^{15}N-**Hby** was eluted in 20 mM HEPES buffer, pH 7.5, containing 100 mM NaCl and 8 M urea. ^{15}N-**Hby** was further purified by RP18-chromatography and crystallized from H_2O/acetonitrile.

HR-MS ^{15}N-Hby (MeOH, 4.5kV): m/z (I [%]) = 875.505 (23), 876.504 (71.1), 877.502 (100, $[C_{45}H_{66}N_8{}^{15}N_2O_8+H]^+$, 878.498 (83.4), 879.496 (47.4), 880.496 (19), 881.494 (6) (see Supporting Figure 3)

Calculated molecular formula of ^{15}N-**Hby**: $C_{45}H_{66}{}^{14}N_{(10-n)}{}^{15}N_nO_8$; n (%) = 0 (10.8), 1 (28.2), 2 (32.1), 3 (20.0), 4 (7.7), 5 (1.2), i.e. average ^{15}N content = 18%

NMR: chemical shift of assignment of ^{15}N (10/10) via 600 MHz ^{1}H and (^{1}H, ^{15}N)-HSQC spectra in H_2O/D_2O 49:1, 10mM sodium phosphate pH 5 (see Supporting Table 1.).

6. Determination of the exchange rates of inner corrin and amide protons by NMR

Determination of the NH exchange rates of the exchangeable protons in **Hby** (c=3.28 mM in 10 mM Na-phosphate buffer pH 5, 2% D_2O) with H_2O were determined at *T*=278, 288, 298, 303, 308, 313 and 318K by 1-dimensional **P**hase-**M**odulated **CLEAN** chemical **EX**change (CLEANEX-PM).[5, 6] The CLEANEX-PM pulse sequence was used from the Bruker standard experiment collection (zgcxesgp) with an excitation sculpting water suppression element.[36] For the determination of the water relaxation times T_1 a saturation recovery experiment was used and the relaxation times ranged between 1.85±0.03 s (R_{1,H_2O} = 0.541 s^{-1}) at 278K to 4.80±0.08 s (R_{1,H_2O} = 0.541 s^{-1}) at 318K. The following experimental parameters were used; spectral width: 19.8 ppm, o1p: 4.7 ppm, number of scans: 512, dummy scans: 2, interscan delay: 2 s. Shaped pulse parameter were set via the "*getprosol*" command and the hard 90° ^{1}H pulse. The mixing times τ_m were set to 10, 20, 40, 60, 100, 140, 180, 250, 300, 350, 400, 600, and 800 ms. The NMR spectra were processed in TOPSPIN 3.2 using a line broadening factor of 2 Hz. The absolute peak areas *A*

of the CLEANEX-PM experiments were used and relative integrals were calculated to the maximal area A_{max} within the τ_{mix}^{-} sequence at a given temperature. The data was the exported to Origin 2016G and the build-up curves were fitted to equation (1)[7] with relative peak area A/A_{max}, exchange rate k [s^{-1}] of NH with bulk water, longitudinal water exchange rate R_{1,H_2O} [s^{-1}], experimental CLEANEX-PM mixing times τ_m [s] and R_{1A}, a combination of the longitudinal and transversal relaxation rate of the NH resonance (floating parameter during fitting).

$$(1) \qquad \frac{A}{A_{max}} = \frac{k}{R_{1A} + k - R_{1,H_2O}} * e^{-R_{1,H_2O}\tau_m} - e^{(R_{1A}+k)\tau_m}$$

7. Crystal structure of Hby by single-crystal X-ray diffraction

Crystals of **Hby** were grown from H_2O/MeCN containing ~10 µM $NaBF_4$ at 5±3°C. Data of a small single crystal of **Hby** were collected at 173K with a Bruker D8 Quest diffractometer (Photon 100 detector) equipped with a microfocus source generator combined with multi-layer optics (monochromatized Mo K_α radiation, λ = 71.073 pm). The structure was solved with SHELXT[8] (version 2014/4) and structure refinement (full-matrix least-squares against F^2) with SHELXL[9] (version 2014/7). Relevant details of the data collection and data evaluation are listed in Supporting Table 2.

Supporting crystallographic data of **Hby** may be obtained from the Cambridge Crystallographic Data Centre CCDC deposition service *via* www.ccdc.cam.ac.uk/structures on quoting the deposition number CCDC 1881269.

7.1. Structural parameters h and ϕ derived from the X-ray crystal structure

The corrin helicity h was calculated as the absolute value of the torsion angle of the virtual dihedron N1-N2-N3-N4. The inter-planar angle ø was derived as the angle between two planes N2-center-N3 and N1-center-N4, were the center corresponds to the central metal ion (or, in **Hby**, to a virtual atom in minimized distance to N1, N2, N3 and N4). The values of h and ϕ were calculated based on the refined X-ray crystallographic data, using mercury 3.9 (Cambridge Crystallographic Data Center) and/or Chem3D 15.0.0.106 (Perkin Elmer).

8. Computational methodology

The initial experimental X-ray crystal structure was utilized as a starting structure for subsequent quantum chemical investigations. **Hby-**tautomers were generated by manual redistribution of hydrogen atoms. **Hby$_{\mathbf{mer}}$(-8)** and **Hby$_{\mathbf{mer}}$(-13)** structures were generated from the most stable BP86/def2-TZVP/D3 cosmo **Hby** conformer. Density functional theory structure optimizations of various **Hby** tautomers were performed with the quantum chemical program suite Turbomole. A def2-TZVP basis set was used for all atoms.[10] All reported calculations were performed with the BP86 [11, 12] density functional in combination with empirical dispersion corrections of the Grimme type with Becke-Johnson damping (BJ).[13] To accelerate the calculations, the resolution-of-identity technique (RI) was used.[14] Solvent effects were treated implicitly by the conductor-like screening model (COSMO) as implemented in Turbomole and a dielectric constant of ε=78.4 was chosen to model water.[15, 16] The influence of other functionals was tested, for example, the PBE density functional.[17] Single point calculations on the BP86/def2-TZVP/D3/cosmo optimized structures yielded very similar results (see Supporting Table 4.). Non-covalent interactions are characterized by a low density and a low reduced density gradient.[18] They can be visualized by an isosurface of the reduced density gradient s, on which sign(λ_2)ρ is plotted, where ρ denotes the electron density and λ_2 is the second eigenvalue of the Laplacian of the density. Its sign can be used to distinguish between various types of interactions: a negative sign is indicative of attractive interactions, such as hydrogen bonds, values close to zero detect dispersion interactions, whereas positive values indicate repulsive interactions.[18] For visualization, the program NCIPlot was used[19] and results visualized with VMD.[20] To assess the conformational flexibility of Co(III), Co(II), and Co(I), amine-cobalt model complexes were set up: an octahedral coordinated Co(III)$(NH_3)_6$, a square-planar coordinated Co(II)$(NH_3)_4$, and a square-planar coordinated Co(I)$(NH_3)_4$ complex. The energy penalty required to increase the inter-planar angle ϕ between the two planes, N1-Co-N3 and N2-Co-N4, from a common plane ($\phi = 0$) were calculated by partial optimization of the structures (BP86/def2-TZVP). The calculated energy penalty at the inter-planar angle $\phi = 13.5°$ (experimental ϕ for **Hby**) was found to be ~13 kJ/mol for $Co^{III}(NH_3)_6$, ~4 kJ/mol for $Co^{II}(NH_3)_4$ and ~6 kJ/mol for $Co^{I}(NH_3)_4$. All calculations were performed with Turbomole[21, 22] and unless mentioned otherwise, structures were visualized with PyMol.[23]

9. Supporting Figures

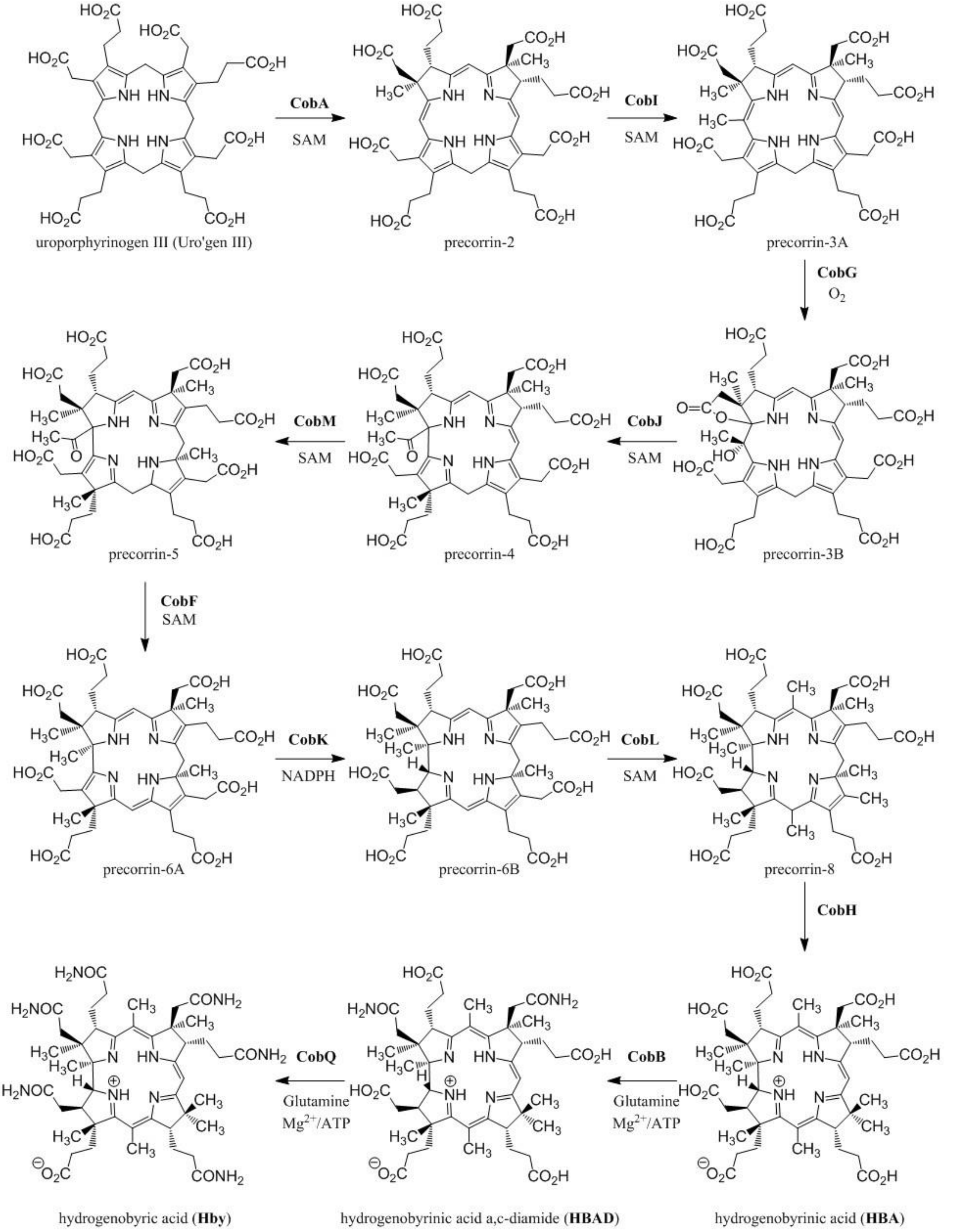

Figure S1. Structural outline of the engineered biosynthesis of hydrogenobyric acid (**Hby**) from uroporphyrinogen III, (**Uro'gen III**) listing critical enzymes CobA-I-G-J-M-F-K-L-H-B-Q and selected essential substrates (adapted from Deery et al., see ref. [2])

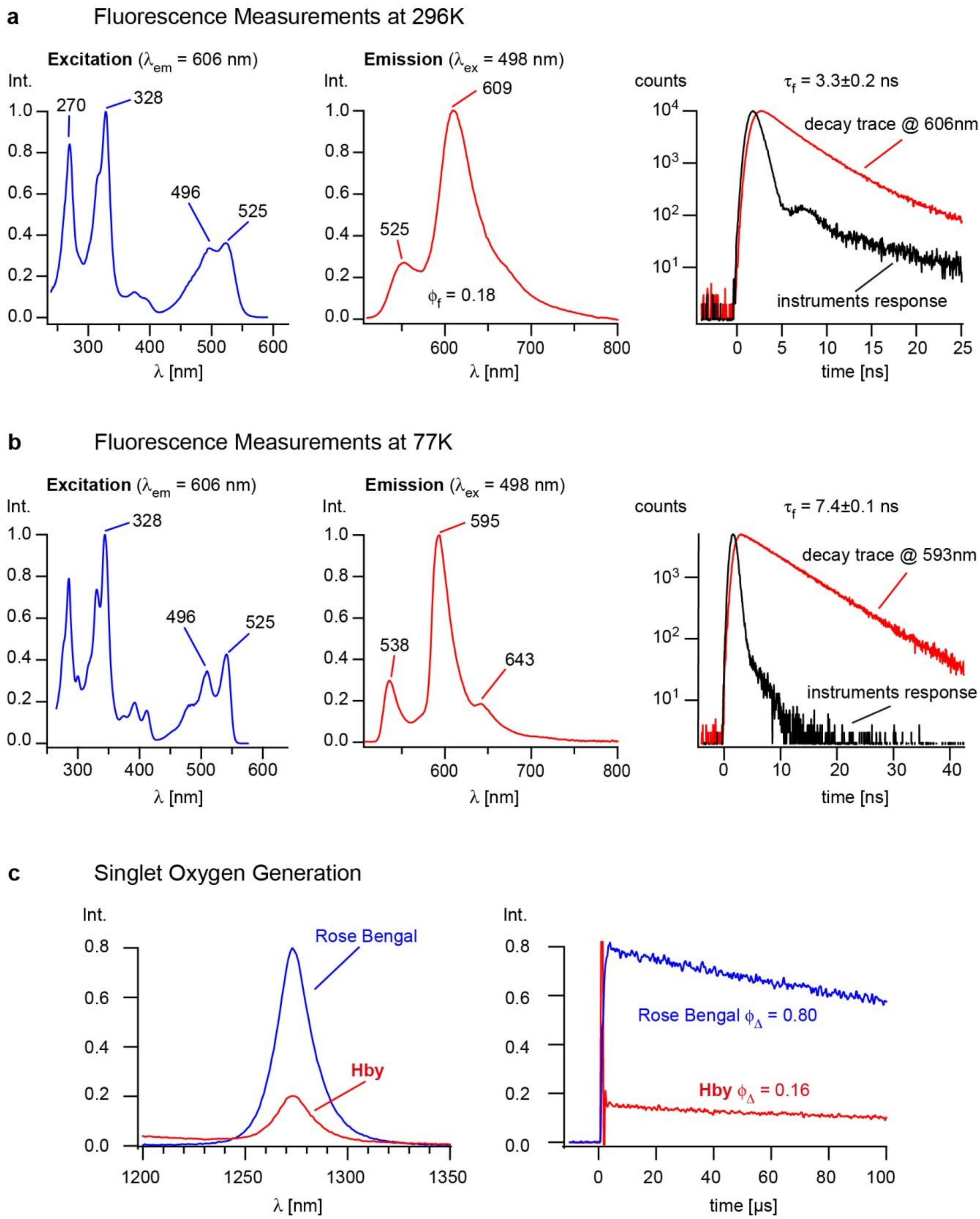

Figure S2. Photo-physical measurements of **Hby** (a, b): Fluorescence excitation (left) and emission (middle) of **Hby** in ethanol at room temperature (a) or at 77K (b). Fluorescence decay traces measured by time-correlated single photon counting (right). (c): Singlet oxygen generation by photosensitization with **Hby** and Rose Bengal, as reference. Singlet oxygen phosphorescence spectra (left) and decay traces (right) of air saturated per-deuterated ethanol solution of **Hby** (red) and Rose Bengal (blue).

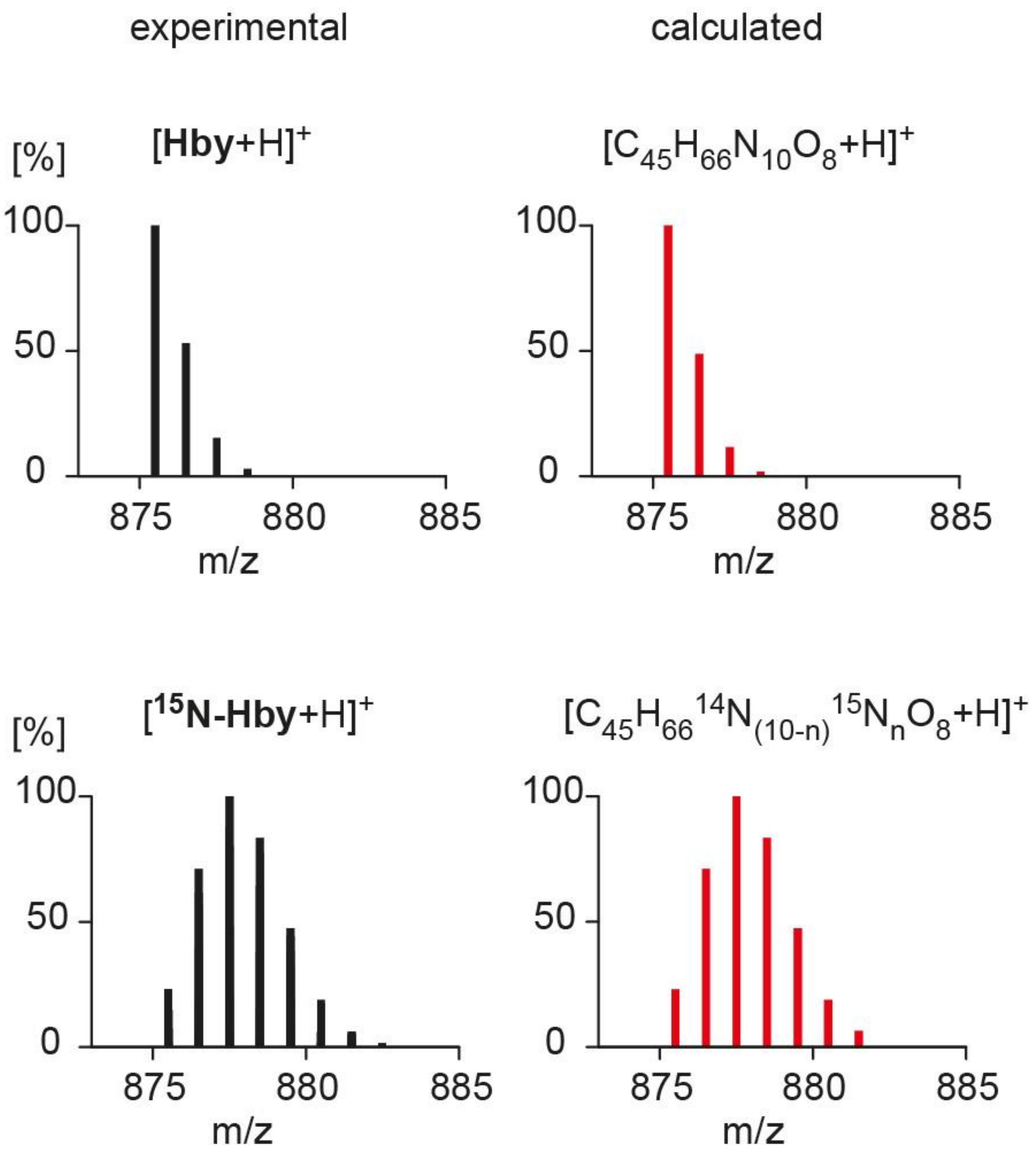

Figure S3. Mass spectrometric analysis of **Hby** (top) and **^{15}N-Hby** (bottom); an average ^{15}N content of 18% is calculated from the mass spectra for the protonated pseudo-molecular ions of **Hby** with natural isotopic abundance $[C_{45}H_{66}N_{10}O_8{+}H]^+$ and of **^{15}N-Hby** $[C_{45}H_{66}{}^{14}N_{(10\text{-}n)}{}^{15}N_nO_8{+}H]^+$ with n (%) = 0 (10.8%), 1 (28.2%), 2 (32.1%), 3 (20.0%), 4 (7.7%), 5 (1.2%).

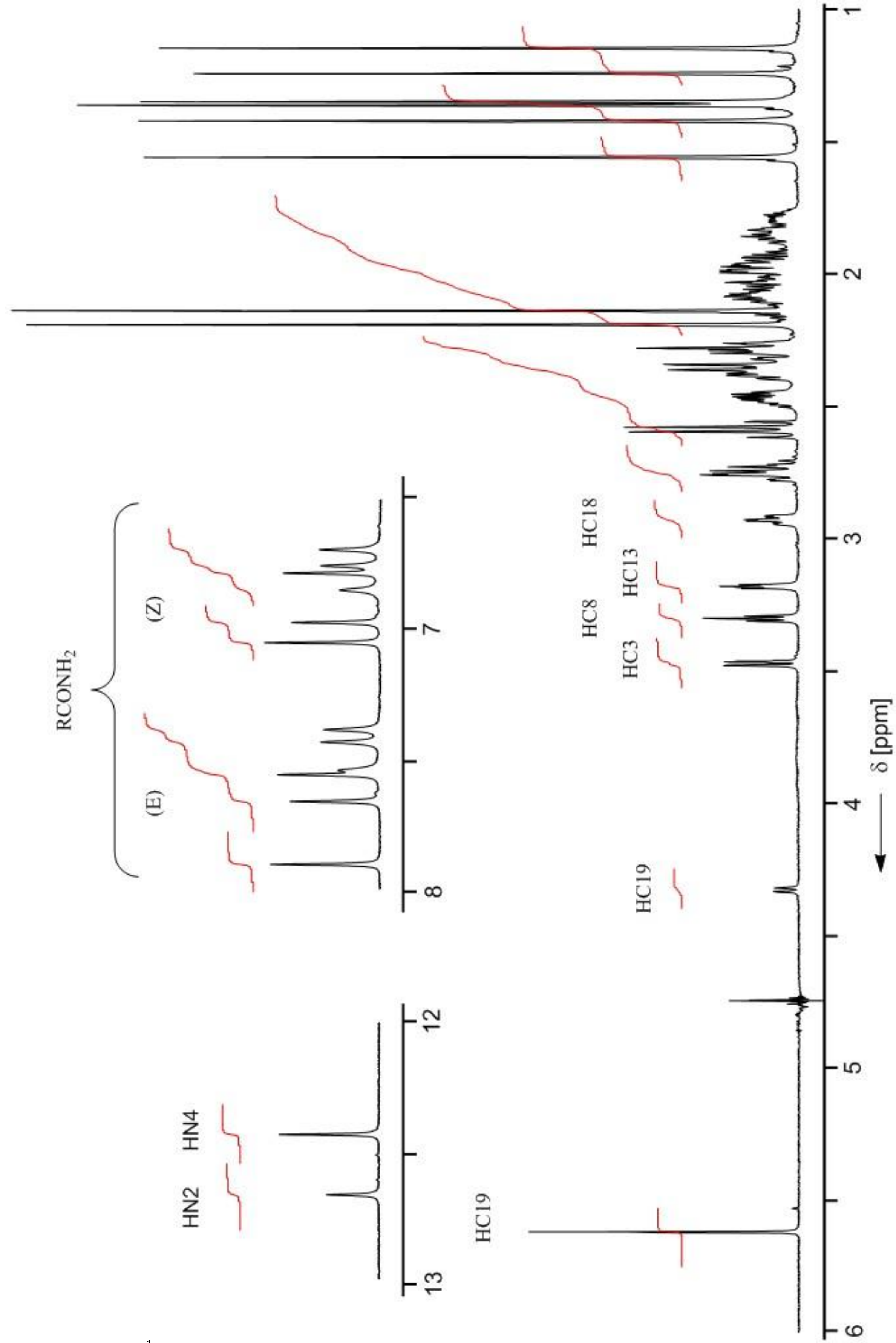

Figure S4-1. 700MHz ^{1}H-NMR of **Hby** in aqueous 10 mM Na-phosphate buffer pH 5, 2%D_2O, c=3.28mM, 298K, H_2O suppression.

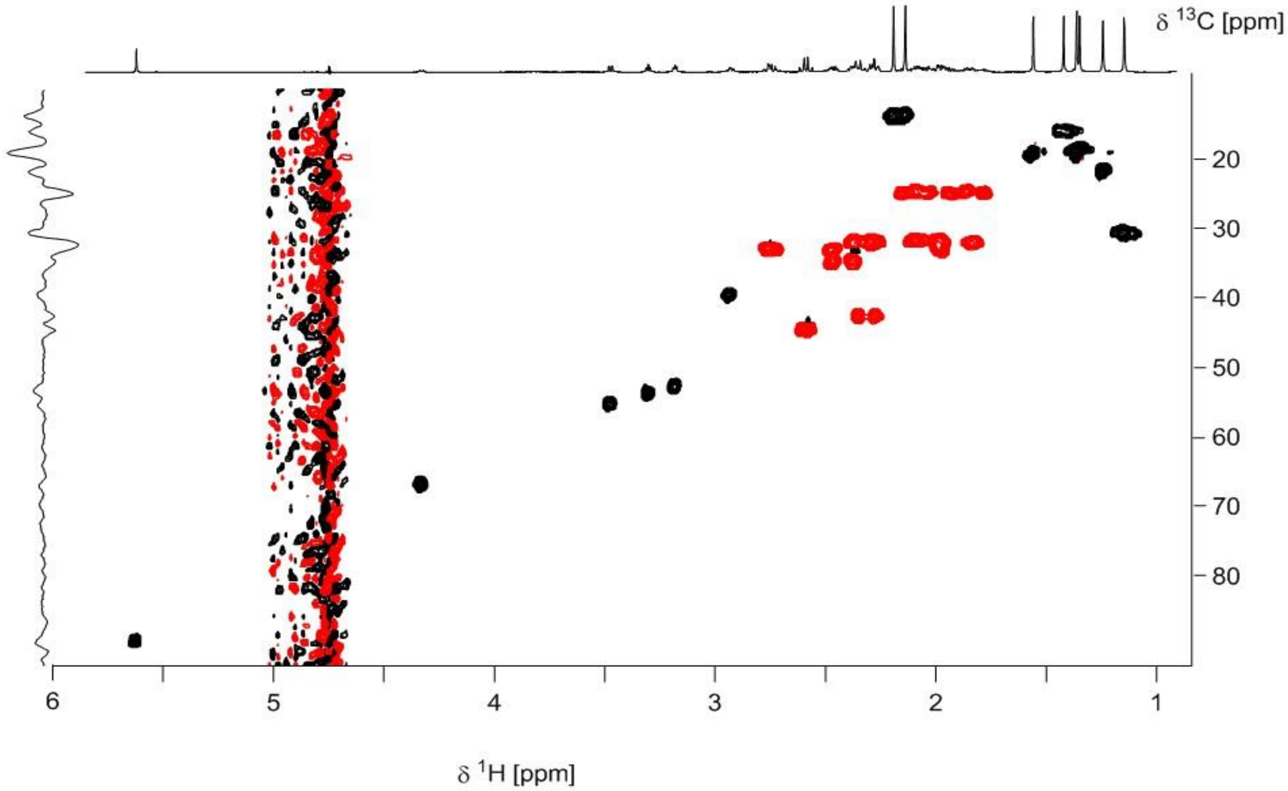

Figure S4-2. 700MHz ^{1}H,^{13}C-HSQC spectrumof **Hby** in aqueous 10 mM Na-phosphate buffer pH 5, 2%D_2O, c=3.28mM, 298K.

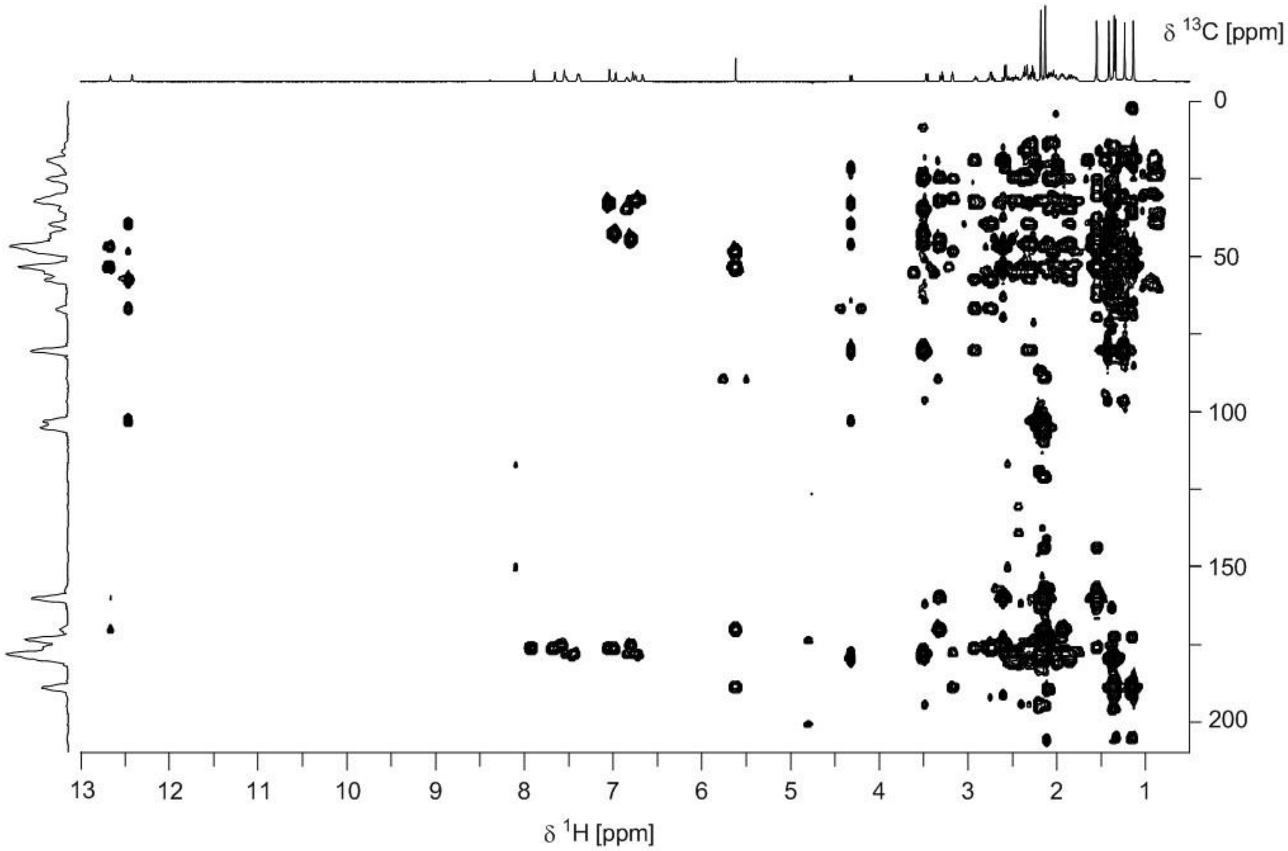

Figure S4-3. 600MHz ^{1}H,^{13}C-HMBC spectrum of **Hby** in aqueous 9 mM Na-phosphate buffer pH 5, 10%D_2O, c=3.14mM, 298K.

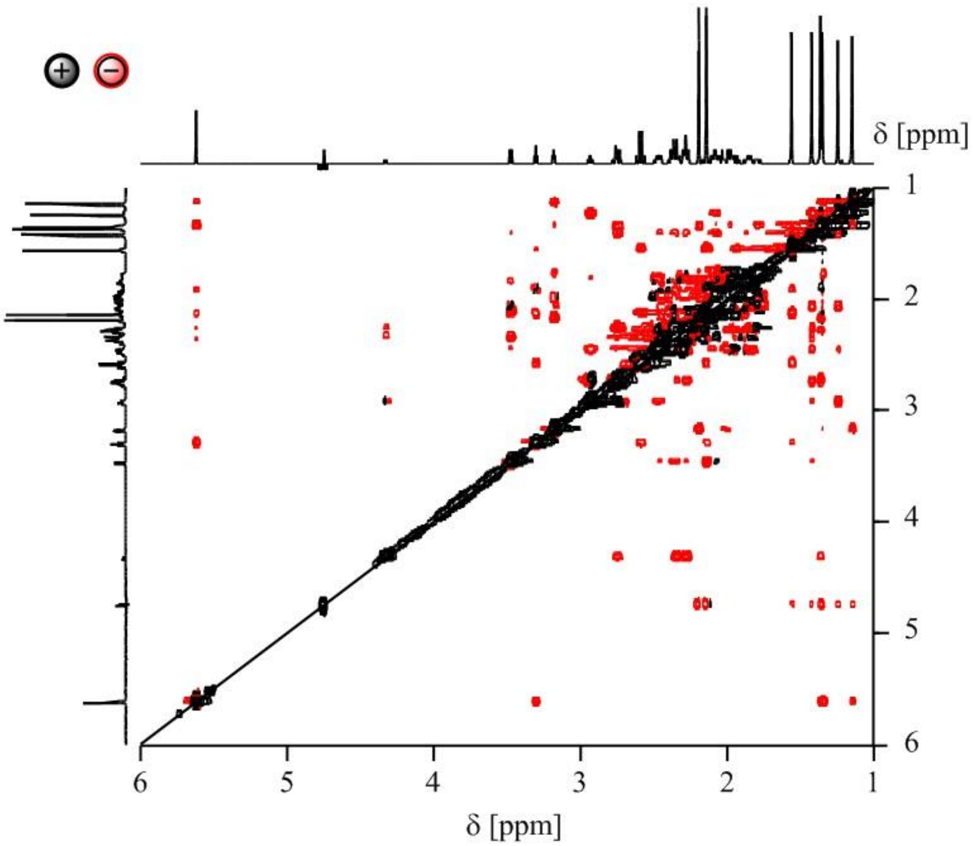

Figure S4-4. High field section of the 700MHz ^{1}H,^{1}H-ROESY spectrum of **Hby** in aqueous 10 mM Na-phosphate buffer pH 5, 2%D_2O, c=3.28mM, 298K.

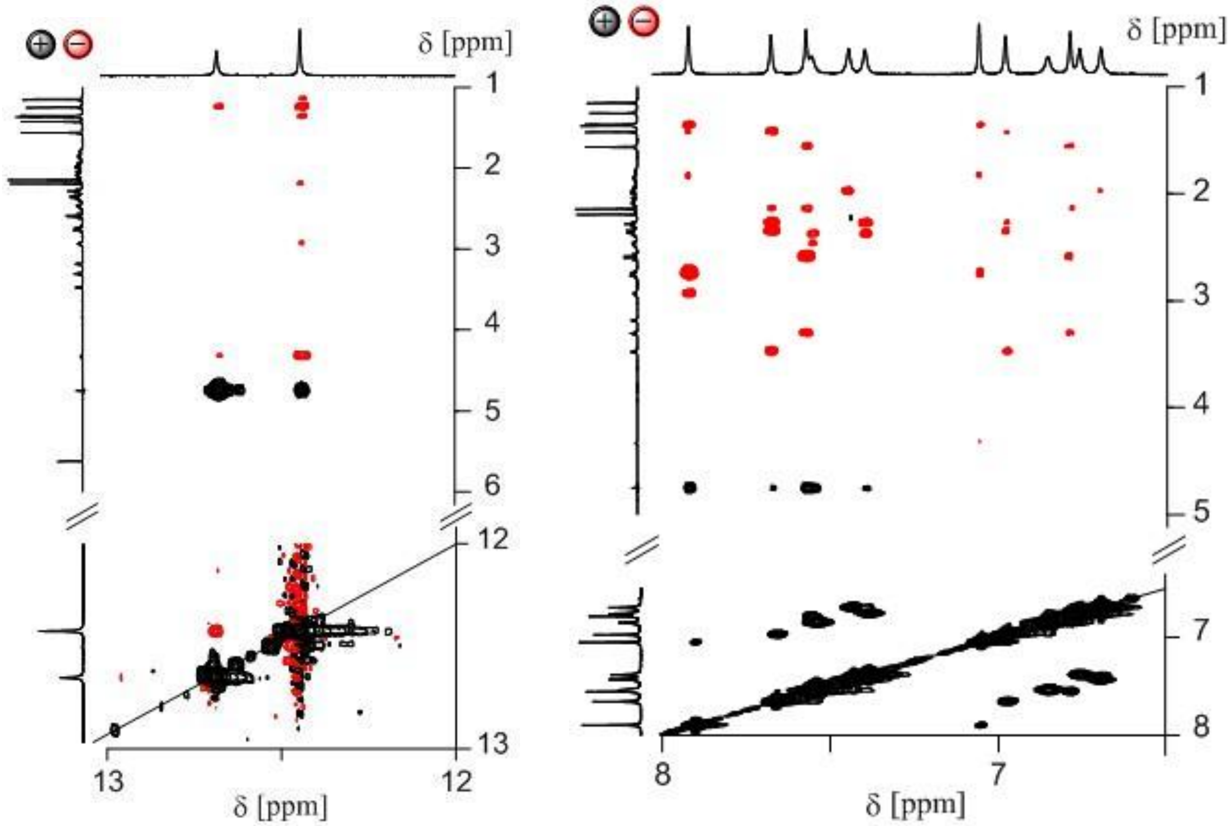

Figure S4-5. Low field sections of the 700MHz ^{1}H,^{1}H-ROESY spectrum of **Hby** in aqueous 10 mM Na-phosphate buffer pH 5, 2%D_2O, c=3.28mM, 298K.

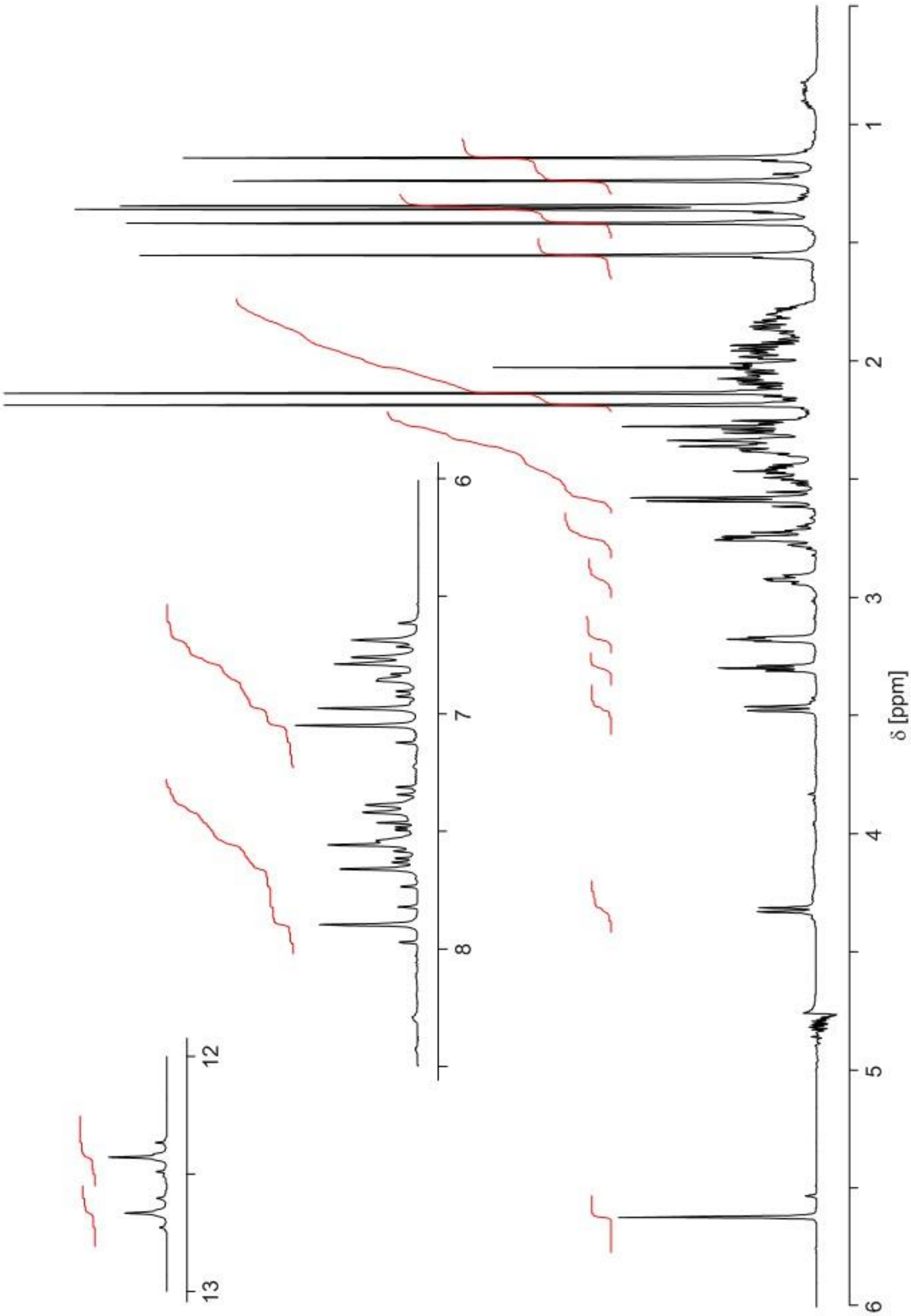

Figure S4-6. 600MHz ^{1}H-NMR of ^{15}**N-enriched Hby** in aqueous 10 mM Na-phosphate buffer pH 5, 2%D_2O, c=5.56mM, 298K, H_2O suppression..

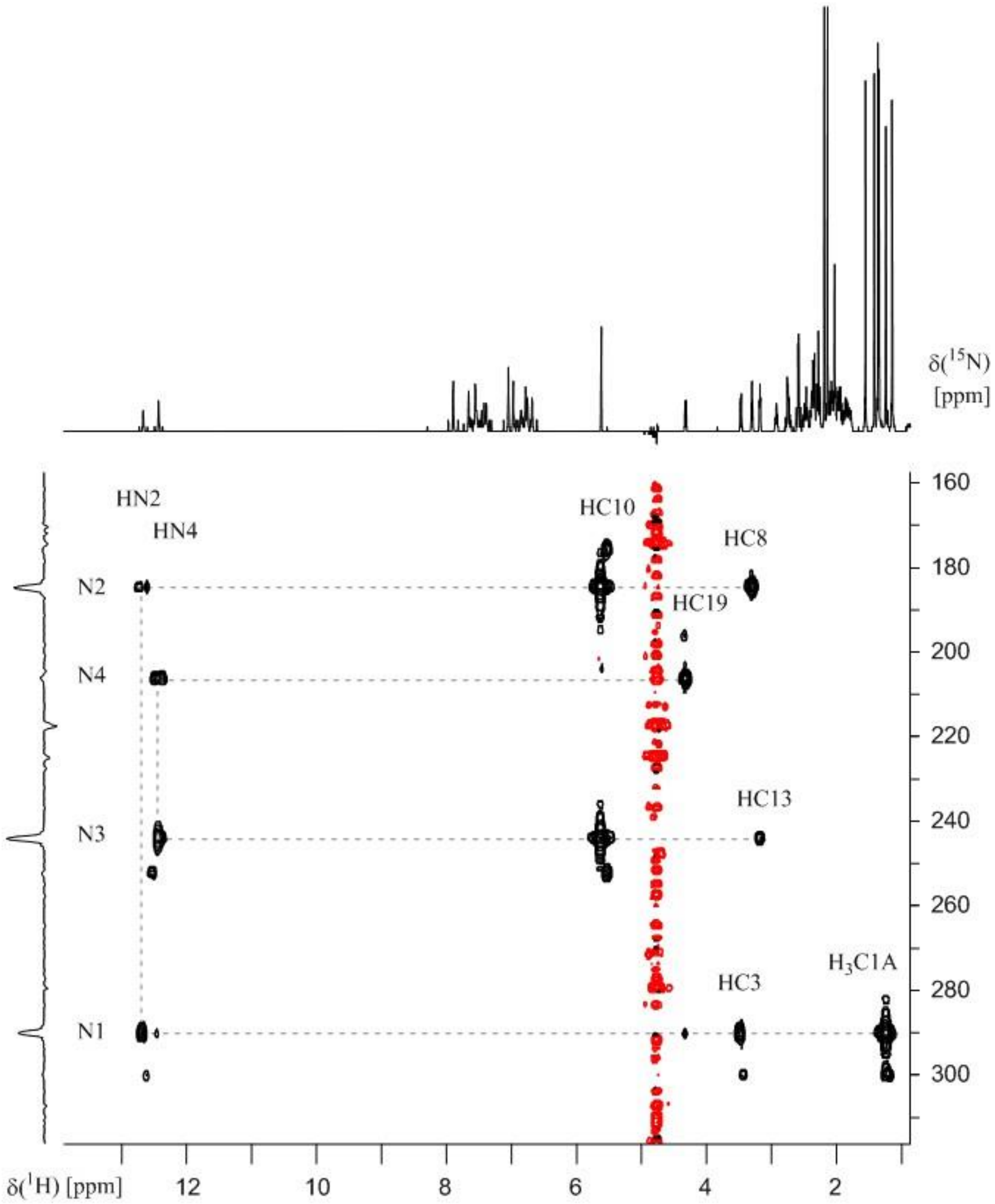

Figure S4-7. 600MHz $^{1}H,^{15}N$-HMBC spectrum of **^{15}N-enriched Hby** in aqueous 10 mM Na-phosphate buffer pH 5, 2%D_2O, c=5.56mM, 298K.

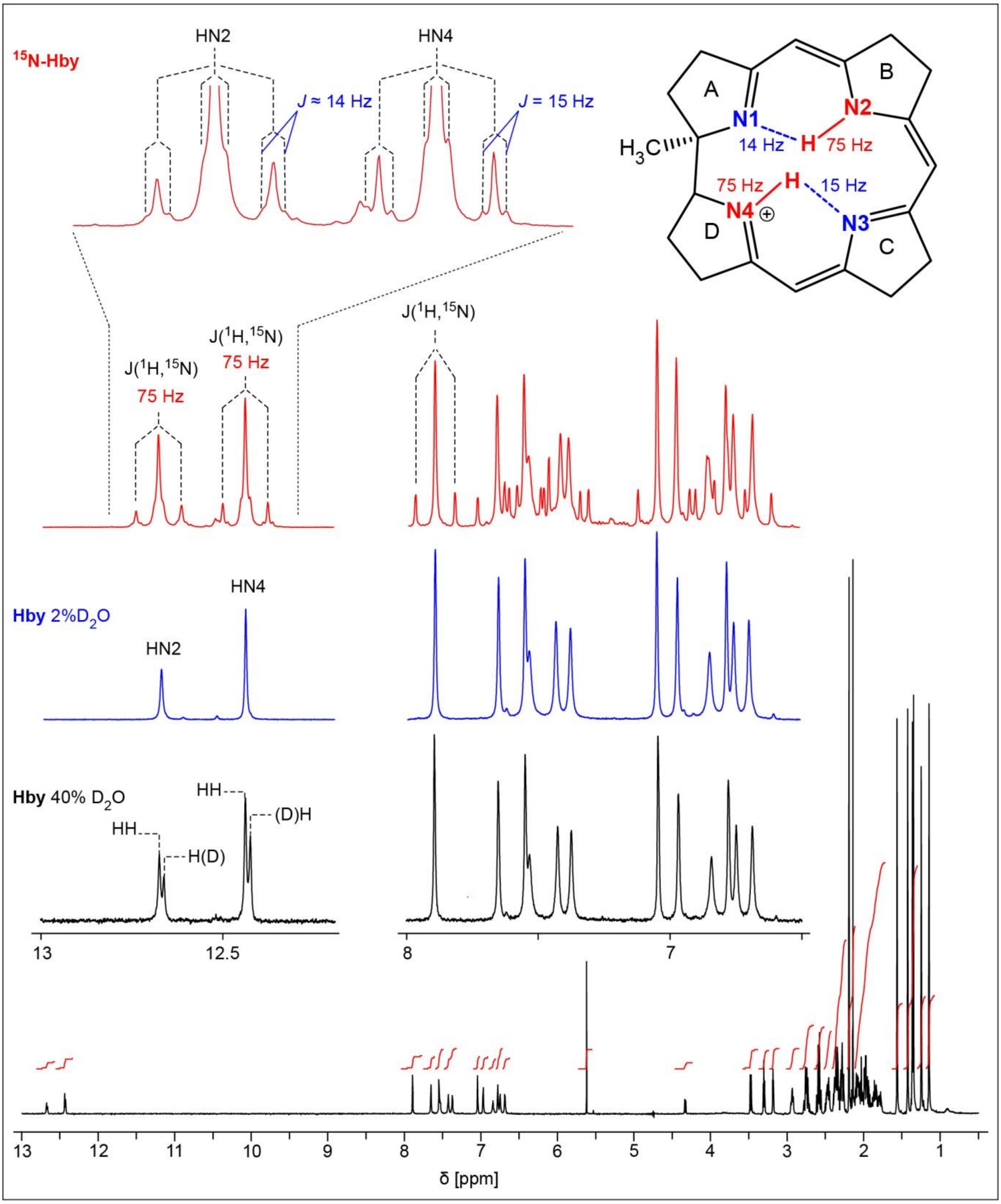

Figure S4-8. NMR spectroscopic analysis of the solution structure of **Hby** in 10 mM Na-phosphate buffer pH 5. 700 MHz ^{1}H-NMR spectrum of an aqueous solution of **Hby** (c=3.28 mM) with 40% D_2O (black) and its expanded low field region, highlighting the effect of one D-atom on the signal position of the remaining H; for comparison, the low field region of a ^{1}H-NMR spectrum of **Hby** in a solution containing 2% D_2O (c=3.28 mM) is shown in blue. Expanded low field regions of a 600 MHz ^{1}H-NMR spectrum of an aqueous solution of ^{15}N-**Hby** (c=5.56 mM) with 2% D_2O (red), featuring heteronuclear ^{1}H, ^{15}N-coupling of the crystallographic H-donor (red) and H-acceptor N-atoms (blue).

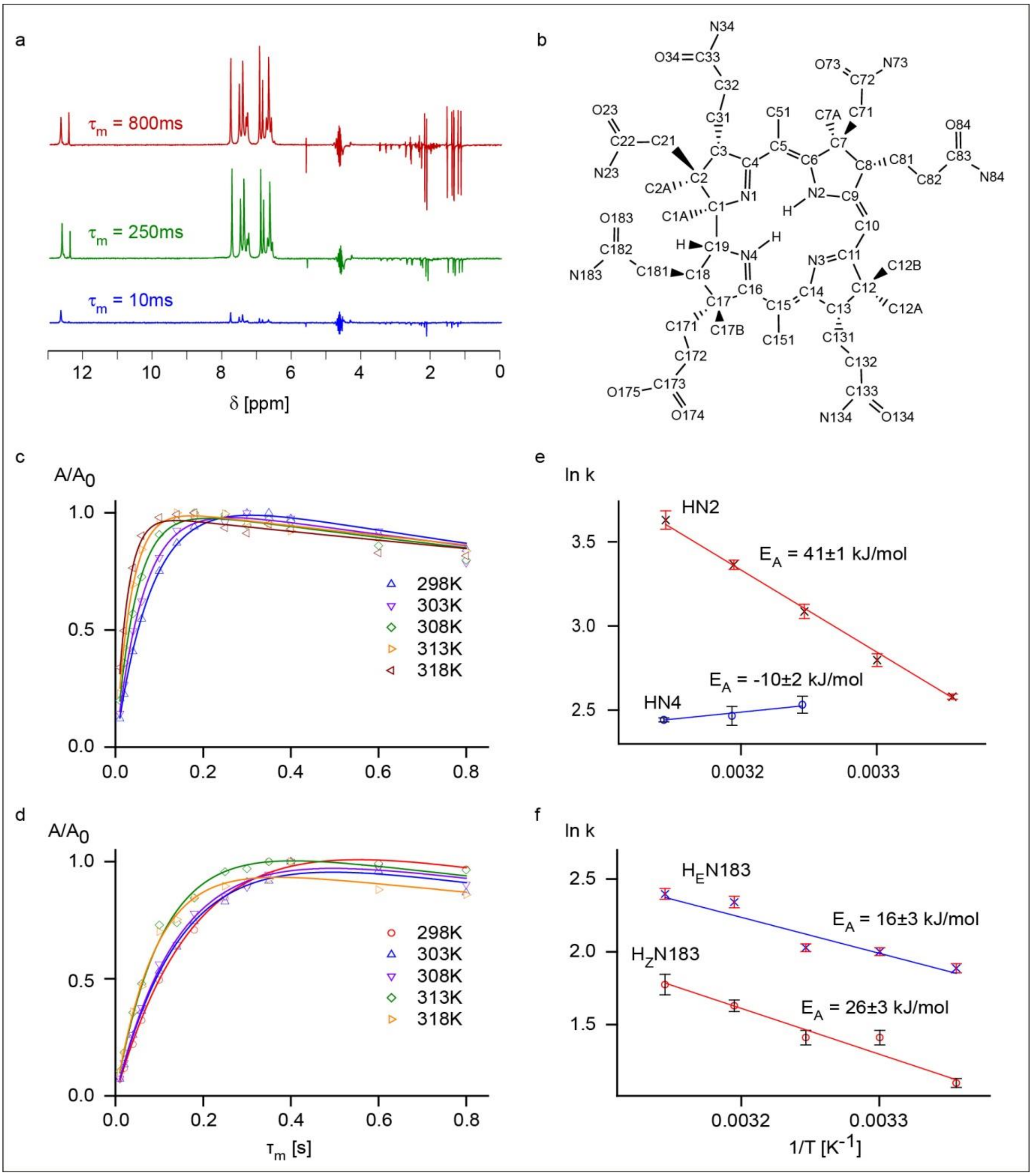

Figure S5. NMR-spectroscopic analysis of exchange of selected H-atoms of **Hby** with solvent H_2O (c=3.28 mM in 10 mM Na-phosphate buffer pH 5, 2% D_2O). (a) Selected 700 MHz ^{1}H-CLEANEX-PM spectra (see refs. [5, 6, 7]) at 318K; (b) atom numbering of **Hby** used for NMR and X-ray crystallography (c) and (d) relative areas of signals for HN2 and for $H_{(E)}$N183, respectively, as function of the experimental mixing time τ_m; (e) and (f) plots of the rates of exchange with H_2O for the 'inner' corrin H-atoms HN2 and HN4, as well as for the peripheral amide protons $H_{E/Z}$N183, used in a kinetic analysis based on the Arrhenius equation. The temperature dependency of the exchange rates for the 'inner' proton HN4 (and the qualitatively different calculated activation energy), compared to the ones of HN2 and the two amide protons at N183, point to a complex mechanism of proton exchange at N4.

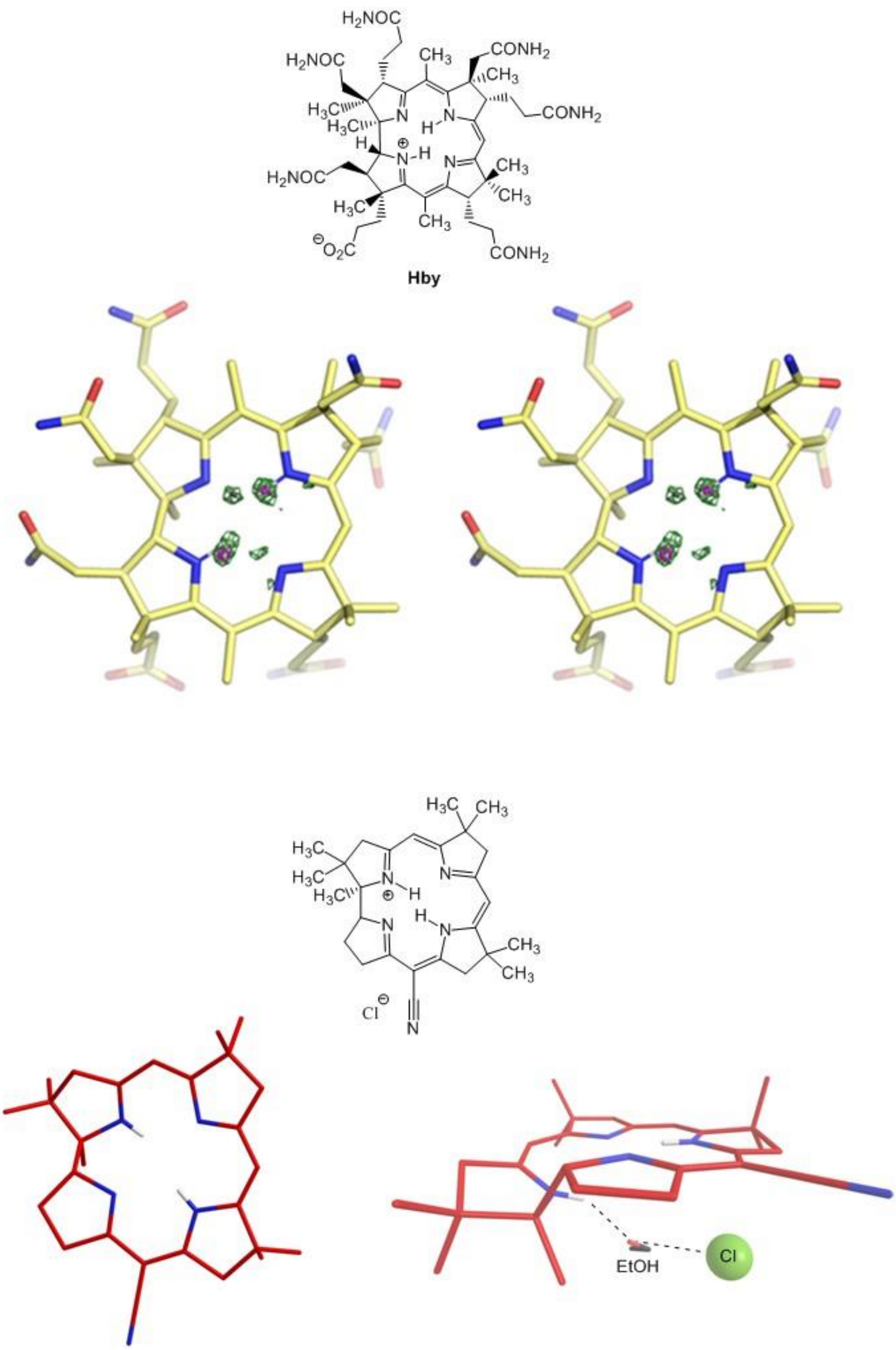

Figure S6. Structural formulae of **Hby** and **HCor**$^+$ and model structures from single crystal X-ray analysis. *Top*. Stereo-picture of **Hby** with highlighted analysis of inner H-atoms of **Hby** from difference electron density, contoured at 3 (green) and 4.5 sigma (magenta), calculated after a refinement with the two hydrogen atoms bonded to N2 and N4 removed from the model. The final crystallographic model of **Hby** is shown in a sticks-representation with carbon colored pale-yellow, nitrogen blue, oxygen red and hydrogen white. *Bottom*. Model structures from X-ray crystal structure analysis of the **HCor**$^+$ chloride ethanol solvate (top view and side view) [24, 25]

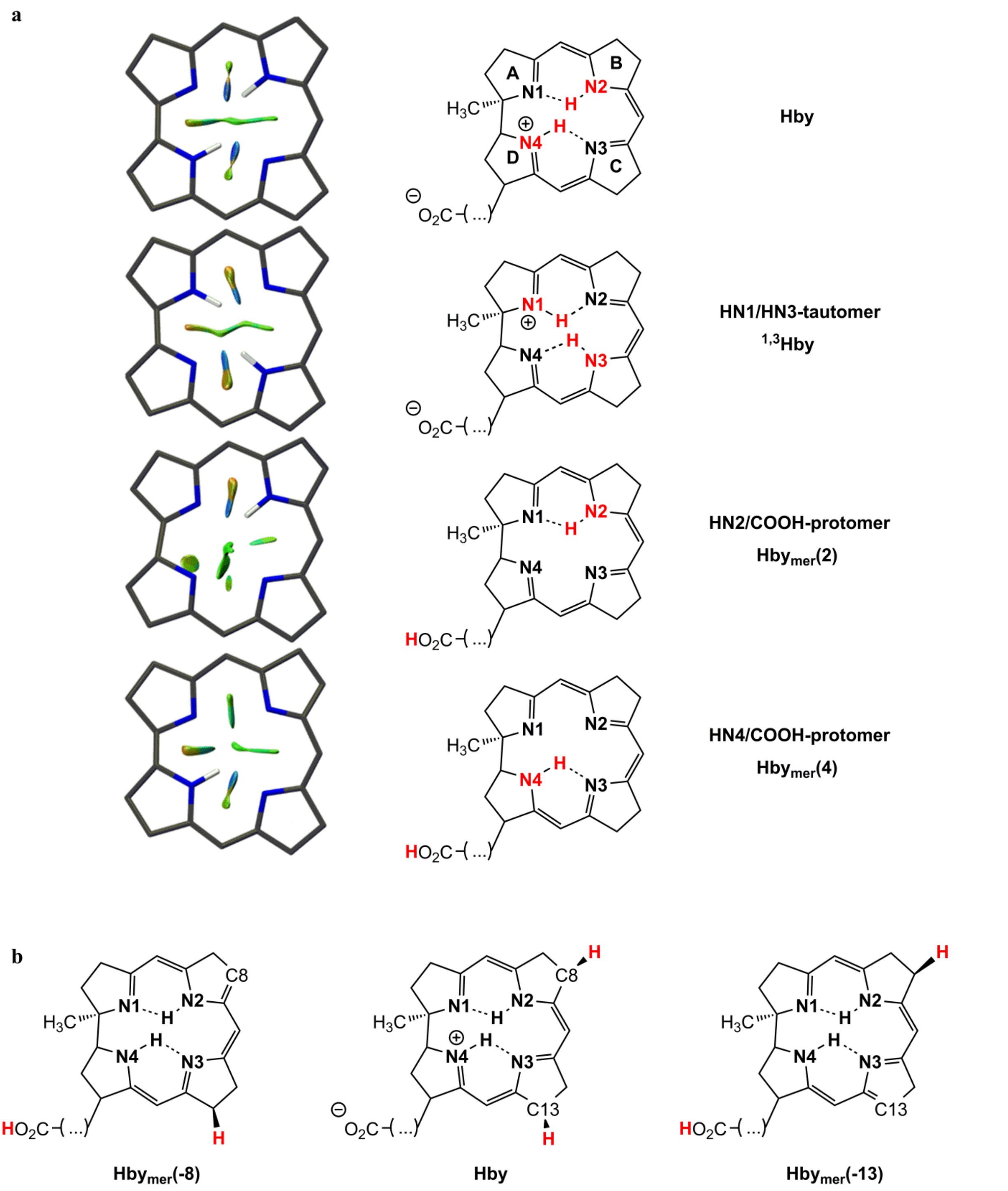

Figure S7. Computational analysis of **Hby**. (**a**) Analysis of non-covalent interactions within the corrin moiety for **Hby**, the ”zwitter-ionic” tautomer **^{1,3}Hby** as well as the “neutral” protomers **Hby$_{mer}$(2)** and **Hby$_{mer}$(4)**, displaying H-bond patterns as visualized by the Lewis formulae. An iso-surface of the reduced density gradient s= 0.5 a.u. is plotted. Blue regions indicate attractive non-covalent interactions, such as hydrogen bonds, green areas dispersion interactions and red repulsive interactions (not indicated relevant in any of these structures). In **Hby** and its tautomer **^{1,3}Hby** strong repulsion between the two opposite hydrogens HN2/ HN4 and HN1/HN3, respectively, is not seen. Rather each H forms an H-bond to the closest of the neighboring nitrogen atoms, as indicated by the dashed lines in the Lewis formulae. (**b**) Lewis formulae of the corrin moiety of **Hby** and of its protomers **Hby$_{mer}$(-8)** and **Hby$_{mer}$(-13)**, in which the proton HC8 or HC13, respectively, has been moved to the f-carboxylate function.

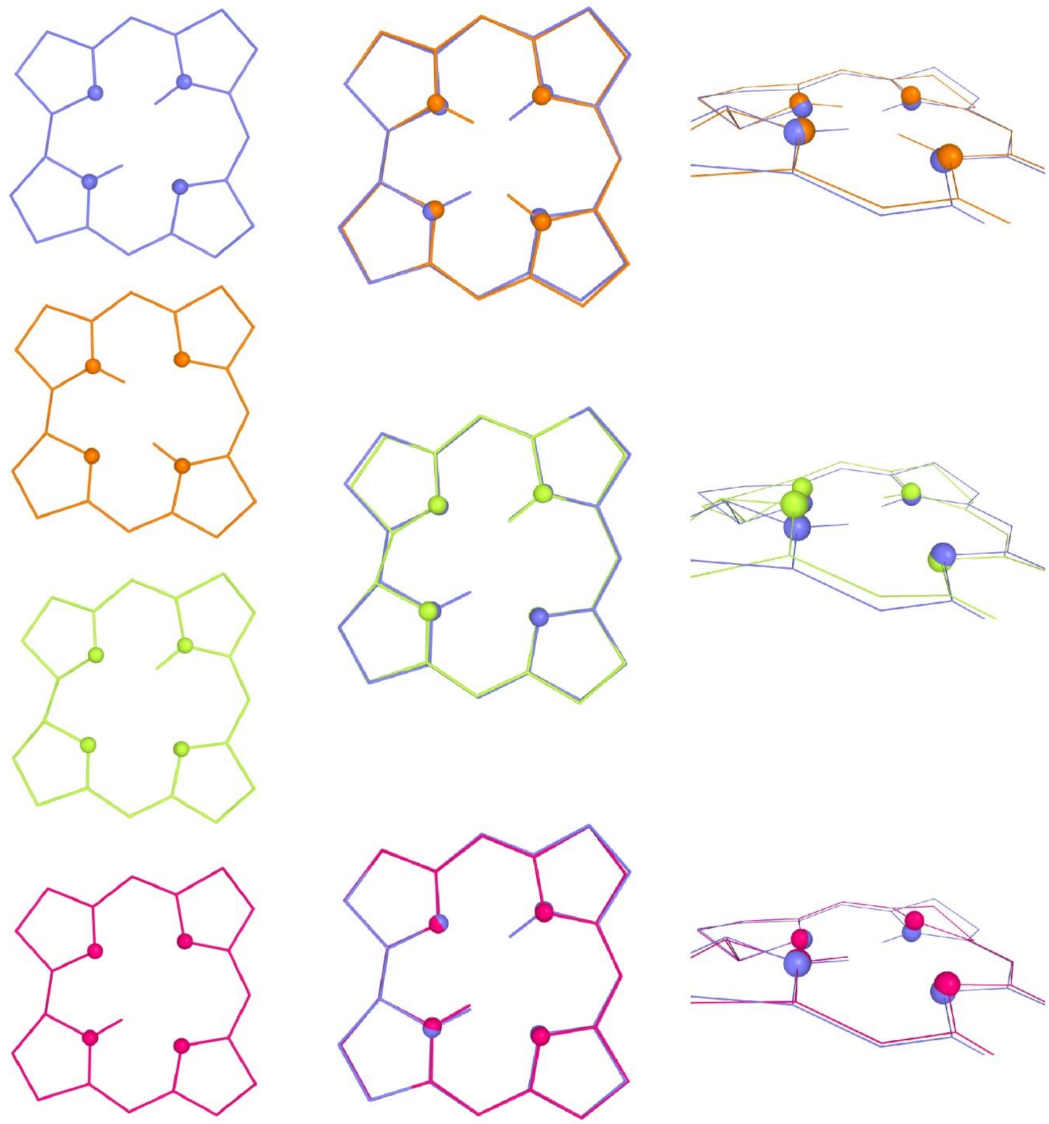

Figure S8. Structural comparison of calculated **Hby** structures. Left column: Core structures of quantum chemically optimized **Hby** (blue), its tautomer **^{1,3}Hby** (orange), and the protomers **Hby$_{mer}$(2)** (lime green) and **Hby$_{mer}$(4)** (magenta) (see Supporting Figure 7 for relevant formulae). Central and right columns. Overlay of calculated core structures. Top: Overlay of calculated core structures of **Hby** and **^{1,3}Hby**, where most notable differences occur at the positions of all four nitrogens, indicating corresponding distortions of the corrin moiety: in **Hby** N2 and N4 are further apart, while in **^{1,3}Hby** N1 and N3 are farther away from each other. Central panel: Overlay of **Hby** (blue) and its protomer **Hby(2)** (lime green), were proton HN4 is moved to the f-carboxylate (see Supporting Figure 7. for formula): the side view (right) displays a further ruffling of the corrin macrocycle at ring D after removal of HN4. Lower panel: Overlay of **Hby** (blue) and its protomer **Hby(4)** (magenta), were the proton HN2 is moved to the f-carboxylate (see Supporting Figure S7): Removal of HN2 also results in a further ruffling of the corrin unit, with a most pronounced effect at ring B and N2. All structures were fully optimized with dispersion and implicit solvent corrections (BP86/def2-TZVP/RI/BJ) and were aligned to the corrin ring atoms of **Hby**.

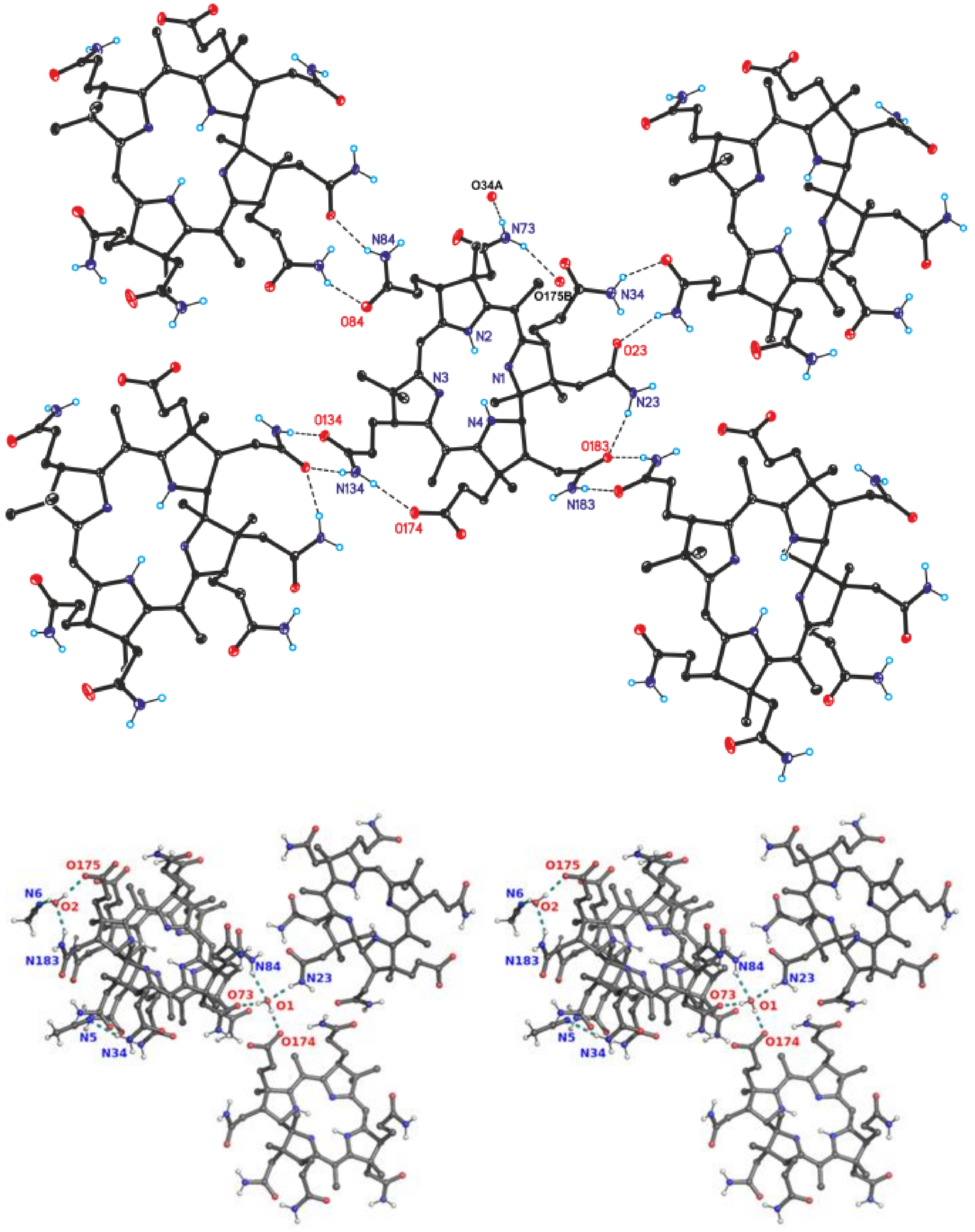

Figure S9. Top. Representative analysis of the H-bonding interactions of an **Hby** molecule in the crystal, pinpointing peripheral groups with their mostly intermolecular H-bonds with neighbouring **Hby** molecules. Bottom. Stereo-picture of a section of the **Hby** single crystal, highlighting the H-bonding pattern of a water molecule surrounded by four molecules of **Hby**, and of two peripheral H-bonding interactions with water and acetonitrile solvent molecules.

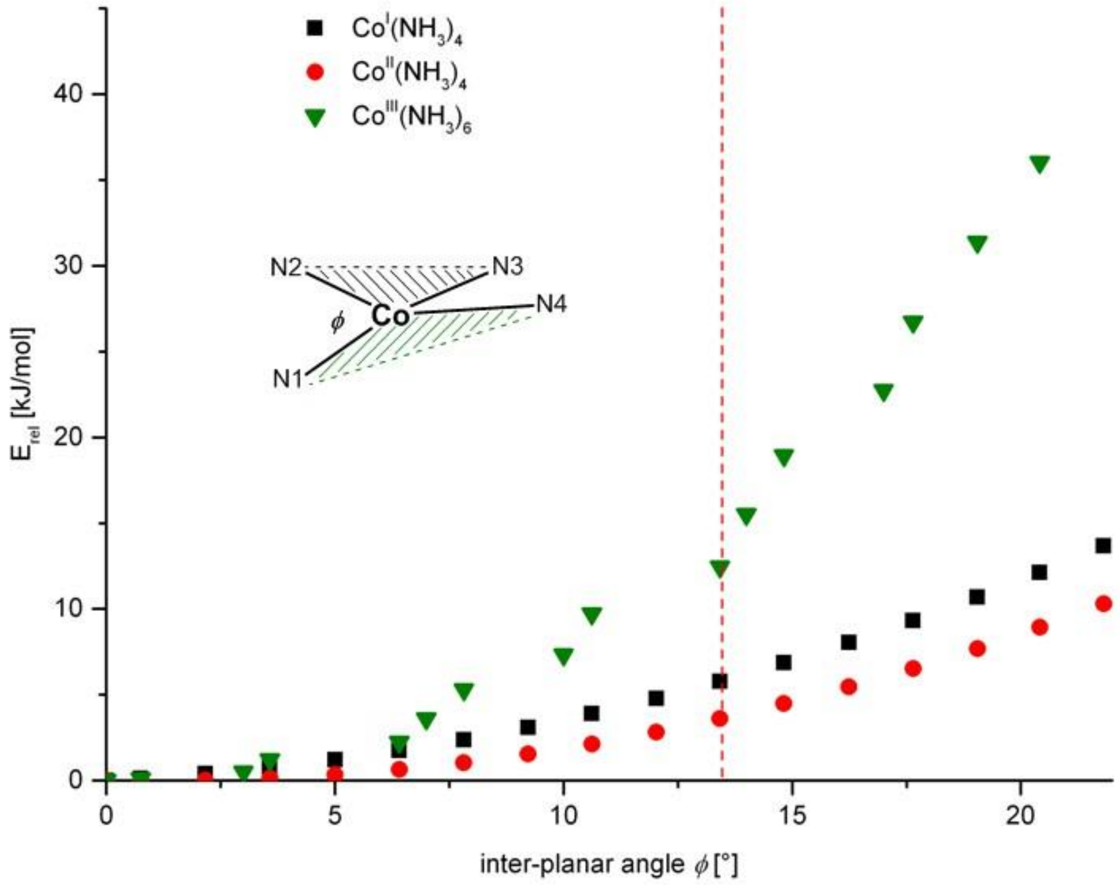

Figure S10. Distortion energy penalties for $Co^{I}(NH_3)_4$ (black squares), $Co^{II}(NH_3)_4$ (red circles) and $Co^{III}(NH_3)_6$ (green triangles) as function of the inter-planar angle ϕ between the planes N2-Co-N3 and N1-Co-N4, from calculated relative energies starting with the most stable (i.e.) square planar, square planar and octahedral arrangements, respectively, at E_{rel} = 0 kJ/mol. The energy penalty associated with an angle ϕ = 13.5° corresponds to the coordination environment for a Co-ion in the undistorted metal free corrin ligand **Hby**. The Co(III)-center features an energy penalty that is strongly increasing with the inter-planar angle ϕ and amounts to roughly 13 kJ/mol for a distortion of ϕ to 13.5°. In contrast, the Co(II)- and Co(I)-centers in the amine complexes can be distorted relatively easy, associated with a calculated energy penalty of only about 4 kJ/mol and 6 kJ/mol, respectively, at ϕ = 13.5°. Hence, for a non-planar arrangement with ϕ = 13.5°, as provided by **Hby**, the transition from the hexa-coordinate Co(III)-complex to a tetra-coordinate Co(II)- or to a tetra-coordinate Co(I)-complex is accompanied by a calculated decrease of the directional strain by about 10 or 7 kJ/mol, respectively.

10. Supporting Tables

Table S1. ^{1}H/^{13}C/^{15}N NMR signal assignments of **Hby** from 600/700 MHz ^{1}H, ^{13}C and ^{15}N NMR-spectra and **^{15}N-Hby** (18 % ^{15}N), in H_2O/D_2O 49:1, 298K. For atom numbering see Figure S5b

atom	δ ^{13}C [ppm]	δ^{15}N [ppm]	δ ^{1}H [ppm]		multiplicity	coupling constants *J*
C1	80.6					
C1A	21.4		1.24		s	
C2	46.3					
C2A	15.6		1.42		s	
C21	42.5		2.28	2.35	AB-system	
C22	176.5					
N23		118.2	6.97 (H_Z)	7.65 (H_E)	s+s	$J(^1H,^{15}N) \approx 89$ Hz
C3	55.1		3.47		d^{app}	$J(^1H,^1H) = 9.5$ Hz
C31	24.4		1.84	2.08	m	
C32	34.3		2.38	2.47	m	
C33	178.4					
N34		112.6	6.85 (H_Z)	7.53 (H_E)	s+s	$J(^1H,^{15}N) \approx 90$ Hz
C4	178.4					
N1		290.1				
C5	105.3					
C51	13.0		2.14		s	
C6	160.0					
C7	47.1					
C7A	18.6		1.56		s	
C71	44.3		2.57	2.61	AB-system	$J(^1H,^1H) = 14$ Hz
C72	175.2					
N73		115.1	6.78 (H_Z)	7.55 (H_E)	s+s	$J(^1H,^{15}N) \approx 90$ Hz
C8	53.4		3.30		t	$J(^1H,^1H) = 6.4$ Hz
C81	24.6		1.91	2.14	m	
C82	32.3		2.29	2.39	m	
C83	178.2					
N84		111.5	6.75 (H_Z)	7.38 (H_E)	s+s	$J(^1H,^{15}N) \approx 90$ Hz
C9	170.2					
N2		184.8	12.67		s	$J(^1H,^{15}N2) = 74.8$ Hz; $J(^1H,^{15}N1) \approx 14$ Hz
C10	89.5		5.62		s	
C11	189.4					
C12	48.6					
C12A	18.0		1.35		s	
C12B	30.4		1.15		s	
C13	52.6		3.18		t	$J(^1H,^1H) = 4.8$ Hz
C131	24.5		1.79	2.00	m	
C132	31.5		2.33	2.42	m	
C133	178.4					
N134		111.6	6.69 (H_Z)	7.34 (H_E)	s+s	$J(^1H,^{15}N) \approx 90$ Hz
C14	177.6					
N3		244.1				
C15	103.1					
C151	13.1		2.19		s	
C16	179.5					
C17	57.5					
C17B	18.9		1.36		s	
C171	31.7		1.85	1.99	m	
C172	32.6		2.30	2.47	m	
C173	180.7					
C18	39.5		2.92		td	$J(^1H,^1H) = 9.5/3.6$ Hz
C181	32.6		2.74		m	
C182	176.2					
N183		114.2	7.05 (H_Z)	7.89 (H_E)	s+s	$J(^1H,^{15}N) = 89.8$ Hz
C19	66.6		4.32		d	$J(^1H,^1H) = 10.3$ Hz
N4		206.4	12.44		s	$J(^1H,^{15}N4) = 74.8$ Hz $J(^1H,^{15}N3) = 15$ Hz

Table S2. Crystal data, structure refinement of **Hby** (CCDC 1881269)

Empirical formula	$C_{45}H_{66}N_{10}O_8$ x 2 H_2O x 2 CH_3CN	
Formula weight	993.21	
Temperature	163(2) K	
Wavelength	0.71073 Å	
Crystal system	Monoclinic	
Space group	$P2_1$ (no. 4)	
Unit cell dimensions	a = 7.0898(3) Å	α= 90°
	b = 24.4617(10) Å	β= 95.064(1)°
	c = 15.2687(6) Å	γ = 90°
Volume	2637.69(19) $Å^3$	
Z	2	
Density (calculated)	1.251 Mg/m^3	
Absorption coefficient	0.089 mm^{-1}	
F(000)	1068	
Crystal size	0.180 x 0.080 x 0.040 mm^3	
Theta range for data collection	2.137 to 25.103°	
Index ranges	$-8 \le h \le 7$, $-29 \le k \le 29$, $-18 \le l \le 18$	
Reflections collected	44438	
Independent reflections	9382 [R(int) = 0.0795]	
Completeness to theta = 25.103°	99.8 %	
Absorption correction	Semi-empirical from equivalents	
Max. and min. transmission	0.971 and 0.932	
Refinement method	Full-matrix least-squares on F^2	
Data / restraints / parameters	9382 / 7 / 671	
Goodness-of-fit on F^2	1.070	
Final R indices [I>2sigma(I)]	R1 = 0.0527, wR2 = 0.0927	
R indices (all data)	R1 = 0.0797, wR2 = 0.1008	
Absolute structure parameter	0.9(5)	
Extinction coefficient	0.0123(11)	
Largest diff. peak and hole	0.221 and -0.218 e.$Å^{-3}$	

Table S3. Crystallographic analysis of hydrogen bonding for 'inner' corrin ring nitrogens HN2 and HN4 of Hby (N_D signifies a formal H-atom donor, H_A an H-atom acceptor), depicting H-bond lengths [Å], pairwise distances between inner corrin ring nitrogens [Å] and H-bond angles [°].

N_D-H···N_A	d (N_D-H) [Å]	d (H···N_A) [Å]	d (N_D,N_A) [Å]	∢ (N_D−H···N_A) [°]
N2−HN2···N1	0.90(3)	1.91(4)	2.619(4)	134(4)
N4−HN4···N3	0.88(3)	2.06(5)	2.665(5)	125(5)
N2−HN2···N3	0.90(3)	2.51(5)	2.996(5)	114(4)
N4−HN4···N1	0.88(3)	2.22(6)	2.608(5)	106(4)

Table S4. Calculated bond lengths [Å] of **Hby** and its tautomer **^{1,3}Hby** as well as of its protomers **Hby$_{mer}$(2)** and **Hby$_{mer}$(4)**, in which H(N4) or H(N2), respectively, are moved to the f-carboxylic function (Figure S7); resulting bond length differences are listed in the three columns at right.

	**Hby**	**^{1,3}Hby**	**Hby$_{mer}$(2)**	**Hby$_{mer}$(4)**	**Δ[Hby-^{1,3}Hby]**	**Δ[Hby-Hby$_{mer}$(2)]**	**Δ[Hby-Hby$_{mer}$(4)]**
N1-N2	2.610	2.616	2.588	2.687	-0.006	**0.021**	**-0.077**
N2-N3	3.014	3.049	3.010	3.016	**-0.035**	0.004	-0.002
N3-N4	2.656	2.624	2.782	2.644	**0.032**	**-0.126**	0.012
N1-N4	2.612	2.632	2.643	2.568	**-0.020**	**-0.032**	**0.043**
N1-N3	3.636	3.865	3.691	3.748	**-0.229**	**-0.055**	**-0.112**
N2-N4	3.989	3.787	4.003	3.882	**0.202**	-0.015	**0.106**
HN1-HN3	-	1.982			-		
HN1-N1	-	1.035			-		
HN1-N2	-	1.811			-		
HN1-N3	-	2.923			-		
HN1-N4	-	2.327			-		
HN3-N1	-	2.887			-		
HN3-N2	-	2.538			-		
HN3-N3	-	1.038			-		
HN3-N4	-	1.830			-		
HN2-HN4	2.058	-			-		
HN2-N1	1.797	-	1.815		-	-0.018	
HN2-N2	1.042	-	1.044		-	-0.002	
HN2-N3	2.466	-	2.454		-	0.012	
HN2-N4	2.978	-	2.974		-	0.003	
HN4-N1	2.258	-		2.198	-		**0.060**
HN4-N2	3.028	-		2.898	-		**0.130**
HN4-N3	1.852	-		1.887	-		**-0.034**
HN4-N4	1.034	-		1.040	-		-0.007
N1-C4	1.298	1.313	1.294	1.294	-0.015	0.004	0.004
C4-C5	1.464	1.428	1.459	1.463	**0.036**	0.005	0.001
C5-C6	1.369	1.395	1.373	1.381	**-0.025**	-0.003	-0.011
C6-N2	1.389	1.381	1.389	1.385	0.009	0.000	0.005
N2-C9	1.346	1.342	1.361	1.323	0.004	-0.015	0.023
C9-C10	1.385	1.403	1.373	1.420	-0.018	0.012	**-0.035**
C10-C11	1.402	1.384	1.424	1.382	0.018	**-0.022**	0.020
C11-N3	1.348	1.351	1.324	1.368	-0.003	**0.024**	-0.020
N3-C14	1.379	1.388	1.395	1.350	-0.009	-0.016	**0.029**
C14-C15	1.398	1.372	1.381	1.415	**0.026**	0.017	-0.017
C15-C16	1.428	1.466	1.469	1.414	**-0.038**	-0.041	0.014
C16-N4	1.317	1.302	1.292	1.330	0.015	**0.025**	-0.013
N4-C19	1.459	1.470	1.463	1.462	-0.011	-0.004	-0.003
C19-C1	1.572	1.572	1.577	1.575	0.000	-0.005	-0.003
C1-N1	1.477	1.464	1.465	1.463	0.013	0.012	0.014

Table S5. Relative stabilities of **Hby** [kJ/mol], of its tautomer **^{1,3}Hby**, and of the **Hby** protomers **Hby$_{mer}$(2)**, **Hby$_{mer}$(4)**, **Hby$_{mer}$(-8)** and **Hby$_{mer}$(-13)** (see Figure S7).

	**gas phase**		**implicit solvent**			
		with dispersion		with dispersion		with dispersion
	BP86/def2-TZVP	BP86/BJ/def2-TZVP	BP86/def2-TZVP	BP86/BJ/def2-TZVP	PBE/def2-TZVP	PBE/BJ/def2-TZVP
**Hby**	0.0	0.0	0.0	0.0	0.0	0.0
**^{1,3}Hby**	39.9	3.96	1.27	22.6	56.1	19.9
**Hby$_{mer}$(2)**	62.6	150.6	74.2	123.3	133.3	118.3
**Hby$_{mer}$(4)**	72.9	153.2	73.4	122.6	135.1	116.7
**Hby$_{mer}$(-8)**				76.3		
**Hby$_{mer}$(-13)**				104.6		

11. References

1. F. J. Widner, A. D. Lawrence, E. Deery, D. Heldt, S. Frank, K. Gruber, K. Wurst, M. J. Warren, B. Kräutler.Total Synthesis, Structure, and Biological Activity of Adenosyl-rhodibalamin, the Non-Natural Rhodium Homologue of Coenzyme B_{12}. *Angew. Chem. Int. Ed.* 2016, **55**(37)**:** 11281-11286. *Angewandte Chemie* 2016, **128**(37)**:** 11451-11456.

2. E. Deery, S. Schroeder, A. D. Lawrence, S. L. Taylor, A. Seyedarabi, J. Waterman, K. S. Wilson, D. Brown, M. A. Geeves, M. J. Howard, R. W. Pickersgill, M. J. Warren. An enzyme-trap approach allows isolation of intermediates in cobalamin biosynthesis. *Nat. Chem. Biol* 2012, **8**(11)**:** 933-940.

3. N. Cadieux, C. Bradbeer, E. Reeger-Schneider, W. Köster, A. K. Mohanty, M. C. Wiener, R. J. Kadner. Identification of the Periplasmic Cobalamin-Binding Protein BtuF of Escherichia coli. *Journal of Bacteriology* 2002, **184**(3)**:** 706-717.

4. E.L. Borths, K.P. Locher, A.T. Lee, D.C. Rees. The structure of Escherichia coli BtuF and binding to its cognate ATP binding cassette transporter. *Proc. Natl. Acad. Sci.* 2002, **99**(26)**:** 16642-16647.

5. T.L. Hwang, S. Mori, A.J. Shaka, P.C.M. van Zijl. Application of Phase-Modulated CLEAN Chemical EXchange Spectroscopy (CLEANEX-PM) to Detect Water−Protein Proton Exchange and Intermolecular NOEs. *J. Am. Chem. Soc.* 1997, **119**(26)**:** 6203-6204.

6. T.L. Hwang, A.J. Shaka. Water Suppression That Works. Excitation Sculpting Using Arbitrary Wave-Forms and Pulsed-Field Gradients. *J Magn Reson., Series A* 1995, **112**(2)**:** 275-279.

7. T.L Hwang, P.C.M.van Zijl, S. Mori. Accurate Quantitation of Water-amide Proton Exchange Rates Using the Phase-Modulated CLEAN Chemical EXchange (CLEANEX-PM) Approach with a Fast-HSQC (FHSQC) Detection Scheme. *J. Biomol. NMR* 1998, **11**(2)**:** 221-226.

8. G.M. Sheldrick. SHELXT - Integrated space-group and crystal-structure determination. *Acta Cryst. Section A* 2015, **71**(1)**:** 3-8.

9. G.M. Sheldrick. Crystal structure refinement with SHELXL. *Acta Cryst. Section C, Structural chemistry* 2015, **71**(Pt 1)**:** 3-8.

10. F. Weigend, R. Ahlrichs. Balanced basis sets of split valence, triple zeta valence and quadruple zeta valence quality for H to Rn: Design and assessment of accuracy. *Phys. Chem. Chem. Phys.* 2005, **7**(18)**:** 3297-3305.

11. A.D. Becke. Density-functional exchange-energy approximation with correct asympototic behavior. *Phys. Rev. A* 1988, **38**(6)**:** 3098-3100.

12. J.P. Perdew. Density-functional approximation for the correlation energy of the inhomogeneous electron gas. *Phys. Rev. B* 1986, **33**(12)**:** 8822-8824.

13. S. Grimme, S. Ehrlich, L. Goerigk. Effect of the Damping Function in Dispersion Corrected Density Functional Theory. *J. Comput. Chem.* 2011, **32**(7)**:** 1456-1465.

14. K. Eichkorn, O. Treutler, H. Öhm, M. Häser, R. Ahlrichs. Auxiliary basis sets to approximate Coulomb potentials. *Chem. Phys. Lett.* 1995, **240**(4)**:** 283-290.

15. A. Schäfer, A. Klamt, D. Sattel, J.C.W Lohrenz, F. Eckert. COSMO Implementation in TURBOMOLE: Extension of an efficient quantum chemical code towards liquid systems. *Phys. Chem. Chem. Phys.* 2000, **2**(10)**:** 2187-2193.

16. A. Klamt, G. Schüürmann. COSMO: a new approach to dielectric screening in solvents with explicit expressions for the screening energy and its gradient. *J. Chem. Soc., Perkin Trans. 2* 1993(5)**:** 799-805.

17. J.P. Perdew, K. Burke, M. Ernzerhof. Generalized Gradient Approximation Made Simple. *Phys. Rev. Lett.* 1996, **77**(18)**:** 3865-3868.

18. E.R. Johnson, S. Keinan, P. Mori-Sánchez, J. Contreras-García, A.J. Cohen, W. Yang. Revealing Noncovalent Interactions. *J. Am. Chem. Soc.* 2010, **132**(18)**:** 6498-6506.

19. J. Contreras-García, E. R. Johnson, S. Keinan, R. Chaudret, J.-P. Piquemal, D. N. Beratan, W. Yang. NCIPLOT: A Program for Plotting Noncovalent Interaction Regions. *J. Chem. Theory Comput.* 2011, **7**(3)**:** 625-632.

20. W. Humphrey, A. Dalke, K. Schulten. VMD: Visual molecular dynamics. *J. Mol. Graph.* 1996, **14**(1)**:** 33-38.

21. R. Ahlrichs, M. Bär, M. Häser, H. Horn, C. Kölmel. Electronic structure calculation on workstation computers: the program system Turbomole. *Chem. Phys. Lett.* 1989, **162**(3)**:** 165-169.

22. TURBOMOLE V6.2 2010, a development of University of Karlsruhe and Forschungszentrum Karlsruhe GmbH, 1989-2007, TURBOMOLE GmbH, since 2007; available from http://www.turbomole.com.

23. The PyMOL Molecular Graphics System, Version 2.0 Schrödinger, LLC.

24. A. Fischli, A. Eschenmoser. A Synthetic Route to Metal-free Corrins. *Angew. Chem. Int. Ed.* 1967, **6**(10): 866-868; *Angew. Chem.* 1967, **79**(19)**:** 865-867

25. E.D. Edmond, D. Crowfoot-Hodgkin. *The Crystal and Molecular Structure of rac-15-cyano-1,2,2,7,7,12,12-heptamethyl corrin hydrochloride, metal free corrin*. Helv. Chim. Acta 1975, **58**(2): 641-654.
